# Interaction of a Porphyrin Aluminum Metal–Organic Framework with Volatile Organic Sulfur Compound Diethyl Sulfide Studied via In Situ and Ex Situ Experiments and DFT Computations

**DOI:** 10.3390/nano13222916

**Published:** 2023-11-08

**Authors:** Shaheed Ullah, Michael L. McKee, Alexander Samokhvalov

**Affiliations:** 1 Department of Chemistry, Morgan State University, 1700 East Cold Spring Lane, Baltimore, MD 21251, USA; 2 Department of Chemistry and Biochemistry, 179 Chemistry Building, Auburn University, Auburn, AL 36849, USA; mckeeml@auburn.edu

**Keywords:** metal–organic framework, diethyl sulfide, sorption, DFT, ATR-FTIR, kinetics

## Abstract

The study presents complementary experiments and quantum chemical DFT computations to reveal the molecular-level interactions of an advanced nanomaterial, porphyrin aluminum metal–organic framework (compound **2**), with the volatile organic sulfur compound diethyl sulfide (DES). First, the intermolecular host–guest interactions during the sorption of DES were explored under dynamic conditions, using the vapor of DES in flowing air. The in situ time-dependent ATR-FTIR spectroscopy in a controlled atmosphere was significantly improved though the use of a new facilely built spectroscopic mini-chamber. The binding site of DES in compound **2** involves the μ(O–H) and COO^-^ groups of the linker of the sorbent. Further, the chemical kinetics of the sorption of DES was investigated, and it follows the Langmuir adsorption kinetic model. That is, depending on the time interval, the process obeys either the pseudo-first- or pseudo-second-order rate law. For the Langmuir adsorption of the pseudo-first order, the rate constant is r_obs_ = 0.165 ± 0.017 min^−1^. Next, the interaction of compound **2** with the saturated vapor of DES yields the adsorption complex compound **3** [Al-MOF-TCPPH_2_]_2_(DES)_7_. The adsorbed amount of DES is very large at 36.5 wt.% or 365 mg/g sorbent, one of the highest values reported on any sorbent. The molecular modes of bonding of DES in the complex were investigated through quantum chemical DFT computations. The adsorption complex was facilely regenerated by gentle heating. The advanced functional material in this work has significant potential in the environmental remediation of diethyl sulfide and related volatile organic sulfur compounds in air, and it is an interesting target of mechanistic studies of sorption.

## 1. Introduction

Metal–organic frameworks (MOFs) are advanced hybrid coordination polymers that have a well-defined nanostructure and feature particularly large nanoporosity. These attractive properties arise from their structure, which contains metal cations, organic functional groups (linkers), and nanopores. Both metals and linkers in MOFs can serve as “anchors” for interactions with molecules. MOFs continue to be a “hot” theme in research and applications, and they have found significant interest in separations and sorption [1], chemosensing [2], heterogeneous catalysis [3], and so on. An important application area of MOFs is the sorption-based deactivation of vapors of hazardous materials and toxic gases, notably industrial chemicals and chemical warfare agents (CWAs) [4].

Aluminum MOFs (Al-MOFs) have attracted great interest for sorption in solution [5] and in gaseous phase [6]. The Al^3+^ cations in Al-MOFs are very stable because they cannot be oxidized or reduced, while certain linkers of Al-MOFs are also stable as anions of carboxylic acids. Many Al-MOFs feature unprecedentedly good stability under corrosive gases, e.g., hydrogen fluoride [7]. Porphyrins are large-ring nitrogen heterocyclic compounds with versatile properties and with multiple application fields in research and emerging technologies. Specifically, porphyrins contain heteroaromatic pyrrole units that favor interactions with polar molecules and aromatic rings suitable for interactions with nonpolar molecules. MOFs with porphyrin linkers were studied as sorbents [8] and chemosensors [9].

**Figure 1:**
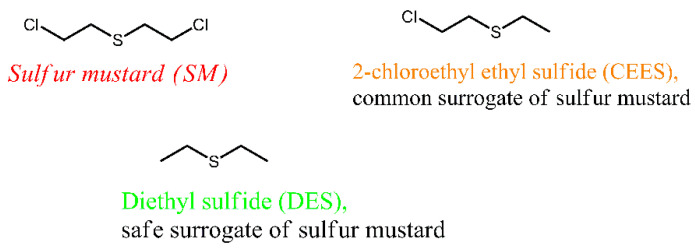
Sulfur mustard and its surrogates with similar structure and properties.

Volatile organic sulfur compounds (VOSCs) have substantial toxicity and a noxious smell, and they are common contaminants of air [10] and water [11]. VOSCs originate in the raw products and exhausts of petroleum and the natural gas industry, and they are also released during the decomposition of organic matter. DES (Figure 1) is a VOSC of simple structure, which is present in products of industrial processing of petroleum and petroleum gas [12] and emitted as an air pollutant from landfills [13] and agricultural facilities [14]. Further, DES finds applications as a solvent in the synthesis of chemicals and in research on hazardous materials; one of the major chemical warfare agents, sulfur mustard (SM), seen in Figure 1, is structurally similar to DES.

Sulfur mustard has the chemical name bis(2-chloroethyl) sulfide, and it can be classified as a halogen-substituted VOSC. Sulfur mustard is extremely toxic, and research of it can only be conducted at specialized military facilities [15]. DES is much less toxic than SM, so it is suitable for university research as a surrogate of SM and potential CWA [16]. Additionally, DES is similar to SM in terms of its physical properties; specifically, it is a volatile liquid (boiling point 92 °C) that creates substantial vapor pressure (60.2 mm Hg) in air.

On the one hand, there are multiple studies on the deactivation of sulfur mustard and its surrogates through chemical oxidation [17], photocatalytic oxidation [18], and specifically photocatalytic oxidation using MOFs [15]. On the other hand, despite the relevance of DES to research on environmental remediation and hazardous materials, studies of its sorption are very rare. Prasad et al. [19] studied the sorption of DES vapor in ambient (moist) air at 36 °C on active carbons impregnated or not impregnated with cations of Ni(II), Cr(VI), Co(II), Cu(II), or Cd(II). Zhang et al. [20] conducted a computational study of sorption of DES and thiophene on Y zeolite using grand canonical Monte Carlo simulation. There are no experimental studies, to our knowledge, of the sorption (or desorption) of DES by any metal–organic framework or related highly porous nanostructured materials such as covalent organic frameworks.

It is of interest to understand the mechanism of sorption of organosulfur compounds [21] and VOSCs as their subgroup to specific binding sites in the sorbent. The investigation of binding sites is often conducted via spectroscopic analysis [22]. In particular, infrared (IR) spectroscopy is very suitable to detect adsorbate molecules in sorbents. Additionally, it allows mechanistic studies of host–guest interactions via shifts of peaks of the host (sorbent) and/or the guest (adsorbate). Attenuated total reflection Fourier transform infrared (ATR-FTIR) spectroscopy uses the evanescent field contacting a solid or liquid specimen on the ATR crystal. When the specimen on the ATR crystal is exposed to vapor and its spectra are recorded, this is referred to as in situ ATR-FTIR spectroscopy. The in situ approach assumes that the controlled environment is created outside of the specimen. The in situ ATR-FTIR spectroscopy has become possible owing to custom-built in situ spectroscopic reactors, starting from a pioneering article by Kazarian et al. [23]. When in situ ATR-FTIR spectra are recorded sequentially at specified time intervals, this is the in situ time-dependent ATR-FTIR spectroscopy, which allows determination of chemical kinetics of sorption or desorption. Surprisingly, there are only very few publications on in situ time-dependent ATR-FTIR spectroscopic studies of sorption of gases or vapors by powders [24]. Recently, we described the in situ time-dependent ATR-FTIR spectroscopy to investigate the mechanisms of sorption and desorption of vapor of water in air on molecular sieves [25] using a custom-built large flow attachment to the FTIR spectrometer.

Quantum chemical simulations synergistically complement the experimental studies. To model interactions of MOFs with molecules, small clusters [26] or the electronic structure theory of periodic solid-state systems [27] are commonly used. Recently, we successfully utilized the DFT method to calculate the geometry and energies of binding sites of aromatic sulfur compounds with clusters of copper MOF [28]. To our knowledge, there are no computational DFT studies of interactions of porphyrin MOFs with molecules.

**Figure 2:**
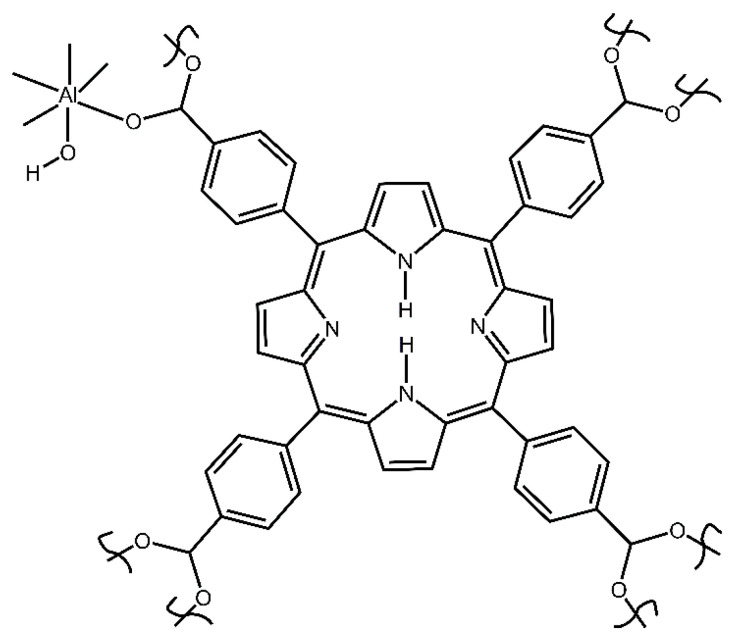
The simplified structural unit of starting compound **2** porphyrin aluminum MOF (Al-MOF-TCPPH_2_).

Herein, we report an investigation of the binding sites of aluminum porphyrin MOF compound **2** (see Figure 2) with DES as vapor in the flowing air, using the in situ time-dependent ATR-FTIR spectroscopy in the controlled vapor atmosphere. A new apparatus was constructed and utilized, a significantly improved spectroscopic mini-chamber ATR-FTIR attachment, which features internal volume of only few cubic millimeters. Also, we determined the chemical kinetics of sorption of DES vapor by compound **2** and investigated the sorption of saturated vapor of DES in the equilibrium (static) conditions. In conjunction, DFT computations were carried out to model the interaction of the DES adsorbate with the MOF. Finally, the reversibility of sorption and desorption was assessed via regeneration of “spent” sorbent.

## 2. Materials and Methods

### 2.1. Chemicals

The first precursor for synthesis of the target material compound **2** (actAl-MOF-TCPPH_2_) was tetrakis(4-carboxyphenyl)porphyrin (abbreviated TCPPH_2_) of ≥97.0% purity from TCI (Portland, OR, USA) and the second precursor was aluminum chloride AlCl_3_·6H_2_O of 99% purity from Thermo Fisher Scientific (Waltham, MA, USA). The solvents for purification of MOF were N,N-dimethylformamide (DMF) of ≥99.5% purity from TCI and acetone of reagent purity from Electron Microscopy Sciences (Hatfield, PA, USA). DES was of >98.0% purity (by GC analysis) from TCI.

### 2.2. Preparation of Activated MOF actAl-MOF-TCPPH_2_ (Compound ***2***)

This compound was prepared and activated as reported earlier by us [29]. Briefly, first, the nonactivated MOF (asisAl-MOF-TCPPH_2_) was synthesized by the autoclave method. Next, the product was thermally activated at 200 °C in the vacuum oven for 21 h. to remove impurities. The so obtained hygroscopic sample was quickly transferred to a jar while it was still in the oven and closed and sealed with Parafilm. The simplified structure of compound **2** is shown in Figure 2. The instrumental characterization of samples is described in Supplementary Materials.

### 2.3. The Hemispherical Gas Flow Spectroscopic Mini-Chamber for In Situ Time-Dependent ATR-FTIR Spectroscopy in the Controlled Atmosphere

**Figure 3:**
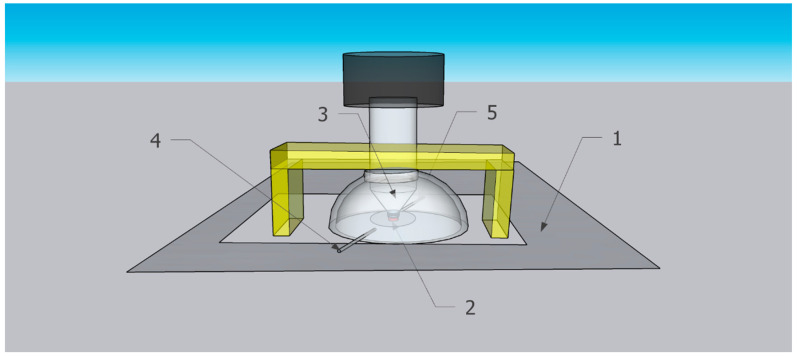
Scheme of spectroscopic mini-chamber installed on baseplate of the FTIR spectrometer with existing ATR assembly.

The home-built hemispherical gas flow spectroscopic mini-chamber attachment in this work is denoted, for convenience, “spectroscopic mini-chamber”. Schematically shown in Figure 3, the spectroscopic mini-chamber was installed on top of the baseplate of the commercial ATR assembly of the FTIR spectrometer Nicolet iS10 (see “Instrumental characterization of samples” in Supplementary Materials). Note that the spectroscopic mini-chamber was placed on top of the spectrometer top baseplate (1, rectangle) which includes the ATR plate with a diamond ATR crystal. The sample (2, red circle) was pressed to the ATR crystal by the ATR anvil (3, gray cone). In Figure 3, item 4 is a gas inlet port while item 5 is a gas outlet port.

In Figure 3, the bridge (yellow) of the ATR assembly is above the spectroscopic mini-chamber and, when engaged, it presses it down. The ATR screw assembly is shown lowered and firmly pressing the specimen to the ATR crystal. The screw of the ATR assembly (white cylinder) protrudes inside the flow chamber via its top opening, and the top screw knob is part of the ATR assembly.

The drawing in Figure 3 was made by the SketchUp Pro 2022 program. The fabrication of the hemispherical gas flow spectroscopic mini-chamber is described in the Supplementary Materials (also see Figure S1).

### 2.4. Dynamic Sorption (in the Flow of DES Vapor) by Compound ***2***, Using In Situ Time-Dependent ATR-FTIR Spectroscopy in the Controlled Atmosphere

The flow of dried air (with RH < 1%) was prepared by the FT-IR Purge Gas Generator (model 74-5041 Parker Balston, from Parker Hannifin Corporation, Haverhill, MA, USA), as described in the section “Instrumental characterization of samples” in Supplementary Materials. The obtained stream of dried air was decreased to a flow rate of 100 mL/min using a dual flowmeter (part 270134.002 from TA Instruments, New Castle, DE, USA) and passed to the inlet of the spectroscopic mini-chamber. Alternatively, for generation of a flow of vapor DES in dried air, a facile in-flow vapor saturation setup was constructed (Figure S2). This setup included a 50 mL Buchner flask equipped with a stopper, which had a glass Pasteur pipette protruding to the bottom of the flask, below the surface of liquid DES (the flask was about half-filled). The flow of dried air was passed through this in-flow setup at a 100 mL/min flow rate, and the obtained stream of DES vapor in dried air was directed to the inlet of the spectroscopic mini-chamber.

Prior to the start of the dynamic sorption experiment, a small sample (few mg) of compound **2** was placed on the ATR crystal. Promptly, the spectroscopic mini-chamber was placed on the ATR base plate (Figure 3), so that its anvil pressed the specimen to the ATR crystal. Then, the locking mechanism of the ATR assembly was engaged, and the sample was ready. First, dried air, prepared as above, was passed through the spectroscopic mini-chamber and a few ATR-FTIR spectra of the intact specimen were collected (as the reference). Each spectrum was averaged 64 times (96 s or ca. 1.6 min). Next, the flow of a gas through the spectroscopic mini-chamber was changed to the purge gas (dried air presaturated with vapor of DES) at the same flow rate of 100 mL/min. Then, the in situ time-dependent ATR-FTIR spectra were collected sequentially, with the same instrumental settings.

### 2.5. DFT Computations

For all geometry optimizations, the Gaussian 16 program [30] was used. The geometry of clusters was prepared by rebuilding the framework of MIL-53(Al), which was reported in our earlier work [31], to incorporate a porphyrin linker TCPPH_2_. The obtained clusters had C_2_ and C_s_ point-group symmetries and were optimized at the B3LYP/6-31G(d)+D3BJ level.

### 2.6. Static Sorption (No Flow) of Saturated DES Vapor in Dried Air by Compound ***2***

Sorption of DES vapor was conducted in a simple vapor saturation chamber, similar to the one described by us [29] for sorption of water vapor. In this work, major modifications were as follows: (a) the vapor saturation chamber did not contain a hygrometer and thermometer, (b) liquid DES was placed inside the chamber to create the vapor, (c) before inserting the specimen, the interior of the vapor saturation chamber was purged with dried air, (d) immediately afterward, the preweighed sample of compound **2** on a quartz XRD specimen plate was inserted into the vapor saturation chamber. The vapor saturation chamber was tightly closed and left at room temperature overnight. The obtained adsorption complex with nominal formula [Al-MOF-TCPPH_2_]_x_(DES)_y_ was denoted compound **3**. Then, compound **3**, when still on a quartz specimen plate, was removed from the vapor saturation chamber, promptly weighed, then returned to the vapor saturation chamber, and stored in it. For an ex situ analysis, compound **3** on a quartz XRD plate was transferred to the specimen chamber of the XRD instrument. Alternatively, a small amount of this compound was placed on the ATR crystal of the FTIR spectrometer.

### 2.7. Desorption of DES Vapor

The “spent” sorbent (adsorption complex) compound **3** was reactivated to remove DES adsorbate. The reactivation was conducted at 30 °C for 72 h in vacuum oven (model AT09e.110, from Across International, Livingston, NJ, USA) connected to a two-stage vacuum oil pump (model RAW-AVP-001, pumping speed 12 cfm, from Xtractor Depot, Oklahoma City, OK, USA). Base pressure in the vacuum chamber was <100 mTorr measured by the Convectron vacuum gauge and digital pressure controller (model 275 Granville-Phillips, from MKS Instruments Inc, Bel Air, MD, USA).

## 3. Results and Discussion

### 3.1. Instrumental Analysis of the Sorbent and Adsorbate

**Figure 4:**
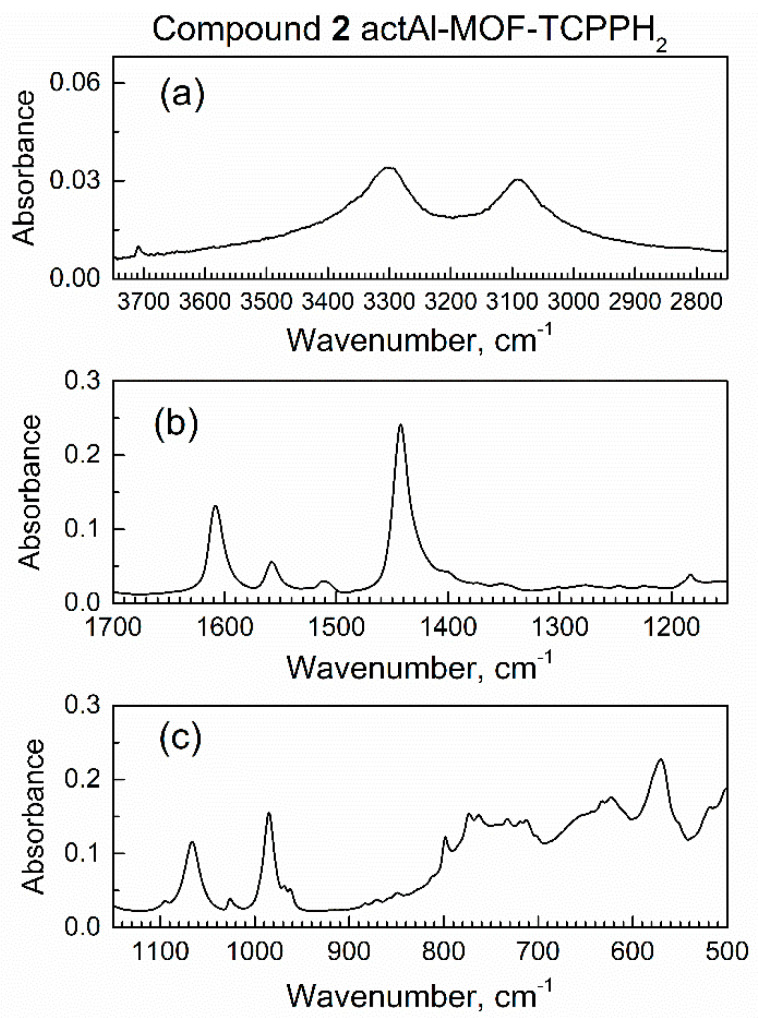
Major ranges of the in situ ATR-FTIR spectrum of compound **2** actAl-MOF-TCPPH_2_ in spectroscopic mini-chamber in flow of dried air: (**a**) high wavenumbers, (**b**) the mid-IR range, (**c**) low wavenumbers range.

Prior to the start of dynamic sorption by the in situ ATR-FTIR spectroscopy, a sample of compound **2** inside the spectroscopic mini-chamber was continuously purged with dried air at a 100 mL/min flow rate. Periodically, the in situ ATR-FTIR spectra were recorded, and the survey spectrum is shown in Figure S3. Figure 4 shows ranges of interest, while peak assignments were provided in our recent work [29].

Within 10 min of purging with dried air, the spectra of compound **2** remained the same, which indicated no leaks of external (humid) air to the spectroscopic mini-chamber.

Figure S4 shows ATR-FTIR and Raman spectra of compound **2** when plotted in the same spectral ranges. Peaks of high intensity in the IR spectrum are of low intensity in the Raman spectrum and vice versa, consistent with selection rules of vibrational spectroscopy. For example, in Figure S4b, the strong Raman peak 1 at 1300 cm^−1^ is present due to nonpolar (but polarizable) vibrations of pyrrole and phenyl in the linker; the corresponding IR peak in Figure S4a is very weak. Next, peak 2, due to the polar COO^-^ group, is very strong in the IR spectrum in Figure S4a but weak in the Raman spectrum in Figure S4b.

**Figure 5:**
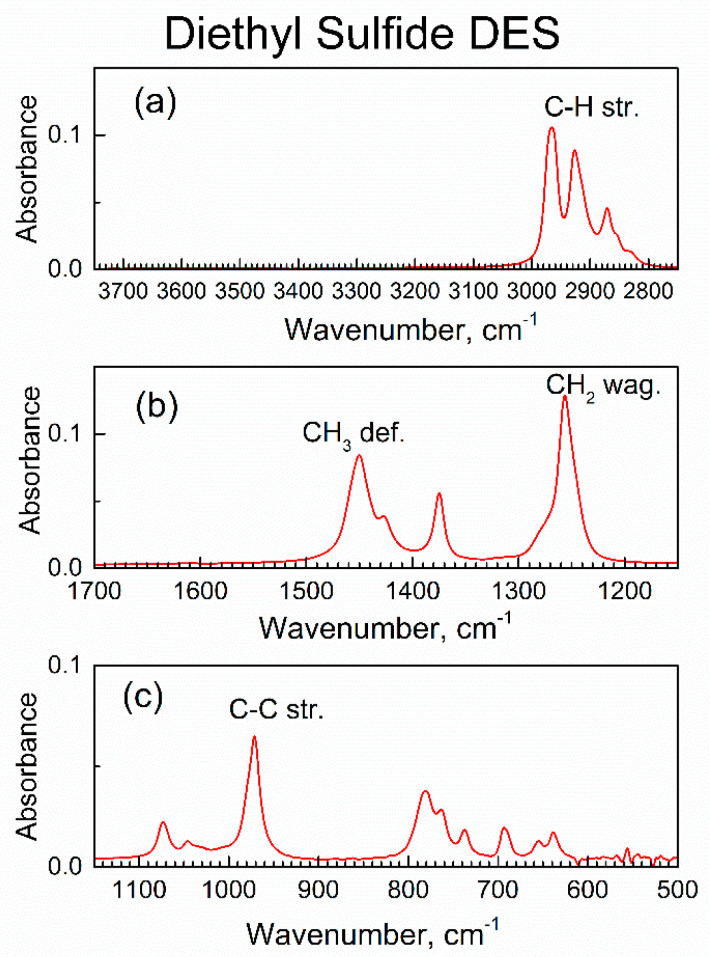
Major ranges of the ATR-FTIR spectrum of liquid DES: (**a**) high wavenumbers range, (**b**) the mid-IR range, (**c**) low wavenumbers range.

Figure S5 shows the survey ATR-FTIR spectrum of liquid DES, the adsorbate in this work. Figure 5 shows the major spectral ranges of the ATR-FTIR spectrum of liquid DES, which are scaled to be the same as for the spectrum of compound **2** in Figure 4.

As expected, the ATR-FTIR spectra of liquid DES in Figure S5 and Figure 5 have the same peaks as published transmission FTIR spectrum [32] of liquid DES. Table S1 shows their assignments [32]; the most characteristic IR peak of DES adsorbate, the C–S stretch at 694 cm^−1^, is of low intensity. Hence, in the study of the interaction of sorbent compound **2** with vapor of DES, the peaks of compound **2** are of importance.

### 3.2. The In Situ Time-Dependent Sorption of DES Vapor by Compound ***2*** Using ATR-FTIR Spectroscopy in Controlled Atmosphere

**Figure 6:**
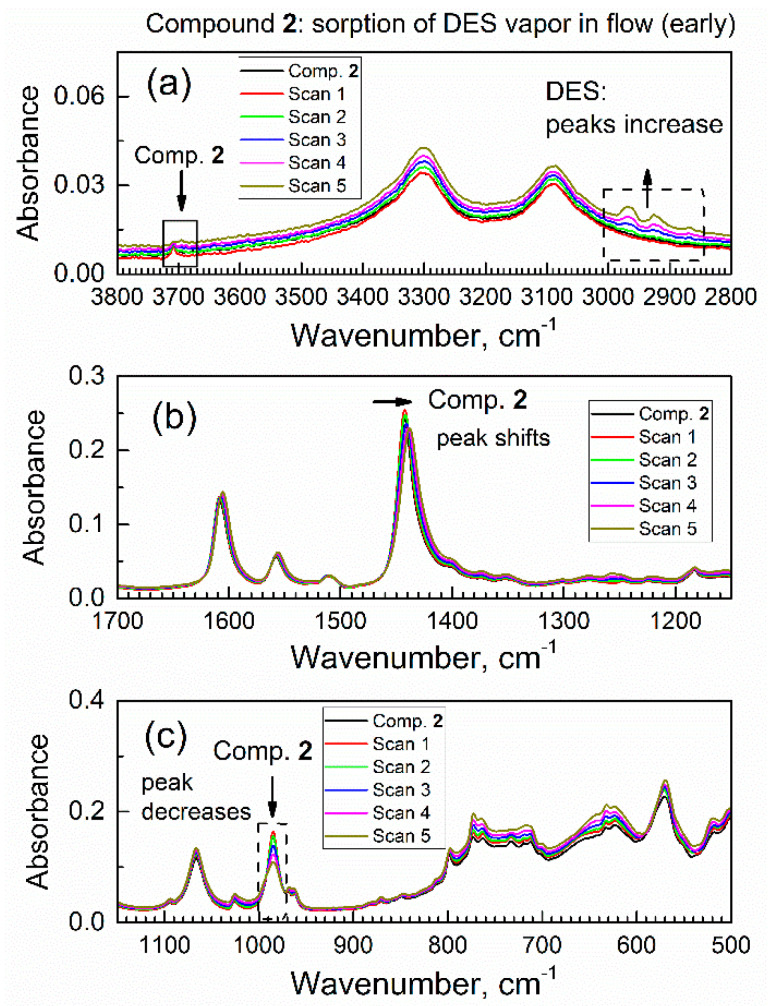
The first set (0–9.6 min) of in situ time-dependent ATR-FTIR spectra of compound **2** in flow of DES vapor in dried air: (**a**) high wavenumbers range, (**b**) the mid-IR range, (**c**) low wavenumbers range.

To our knowledge, there are no reports of sorption of DES on any MOF. After collecting the ATR-FTIR spectrum of compound **2** in a flow of dried air through the spectroscopic mini-chamber, the flow of gas was changed to the purge gas (i.e., dried air saturated with DES vapor). Under the flow of the purge gas, the in situ ATR-FTIR spectra were periodically collected (see Figure 6); one spectrum takes 64 scans (1.6 min). One can see gradual changes in spectra which accumulated with time, and the observed spectral changes were of two kinds. On the one hand, there is an increase in absorbance of peaks within ca. 3000–2850 cm^−1^ (marked with an upward arrow and dashed square frame). Namely, these growing peaks are at 2967, 2922, and 2867 cm^−1^ and they belong to vibrations of the DES molecules (see Table S1).

Specifically, these peaks were due to the antisymmetric stretching vibration of the CH_2_ group, the symmetric stretching vibration of the CH_2_ group plus the stretching vibration of the CH_3_ group, and the symmetric stretching of the CH_3_ group. The growth of these peaks indicated a progressive increase in the amount of DES absorbed by compound **2** from the purge gas. On the other hand, there were several concurrent shifts of peaks of specific functional groups of compound **2**; this indicated that DES adsorbate interacted with those groups. First, in Figure 6a, the peak at 3708 cm^−1^ (marked with a downward arrow and a square frame) due to the stretch vibration of a free O–H group in compound **2** underwent a progressive decrease.

Second, in Figure 6b, there is a red shift of peak at 1442 cm^−1^ due to the COO^-^ carboxylate group in sorbent compound **2**; the magnitude of this shift exceeded the nominal resolution of FTIR spectrometer. Additionally, this peak not only shifted, but also became lower in absorbance at its maximum and wider. These changes were indicative of interaction of the COO^-^ carboxylate group in compound **2** with DES adsorbate. Third, in Figure 6c, one can see a progressive and significant decrease of the peak at 985 cm^−1^ due to vibration of the μ(O–H) group connected to aluminum atom in compound **2**. This decrease was quite significant, at about 1/3 of the initial IR absorbance. In contrast, the next peak at ca. 1067 cm^−1^ only underwent minor drift together with spectral baseline. For some specimens, the change of contact with the ATR crystal could occur; namely, certain materials can “swell” upon sorption of adsorbate vapor [33], which could affect IR absorbance. However, this artifact would affect all peaks via the same type of spectral change, and this was not observed. These observations were consistent with a sorbent–adsorbate interaction.

Overall, the diverse and selective changes of the IR peaks of compound **2** in Figure 6 during the contact of compound **2** with DES vapor indicate that changes only occurred with certain functional groups in compound **2** (that interact with DES molecules).

Figure S6 showed the second set of in situ ATR-FTIR spectra (labeled6–11), collected sequentially during the time of 9.7–19.2 min. In Figure S6a, peaks due to DES adsorbate continued to grow until about scan 8, and then stabilized. The observed IR absorbance at peak maximum was higher, when the amount of the respective compound in the specimen (sorbent) was larger. This indicated that the highest adsorbed amount of DES was reached by scan 8 (namely, by 8 × 1.6 min = 12.8 min). At the same time, in Figure S6c, the peak at 985 cm^−1^ due to the μ(O–H) group of compound **2** was decreasing until scan 8, and then it became constant. The correlation between the increase of peak due to DES adsorbate and the decrease of the peak due to a specific functional group in sorbent (compound **2**) meant that the DES adsorbate interacted with the μ(O–H) group in compound **2**. Other peaks (Figure S6b) changed to a much lesser extent, which indicated strong interaction of DES with the μ(O–H) group.

After about 30 min of flow of DES vapor, the ATR-FTIR spectra remained the same; an attempt was made to regenerate “spent” sorbent under the in situ conditions to its active form (without DES adsorbate). Here, after about 40 scans (64 min), the gas flow was changed to dried air, and the intensity of the IR peaks of the DES adsorbate in the specimen had somewhat decreased but did not reach zero. It indicated that regeneration of “spent” sorbent could not be achieved under the in situ conditions; the ex situ regeneration was described in one of the subsequent sections. This was of interest to quantitatively analyze the dynamics of in situ changes of peaks due to DES adsorbate (see below).

### 3.3. Time Progression of In Situ Sorption of DES Vapor by Compound ***2*** and Analysis of Its Chemical Kinetics

**Figure 7:**
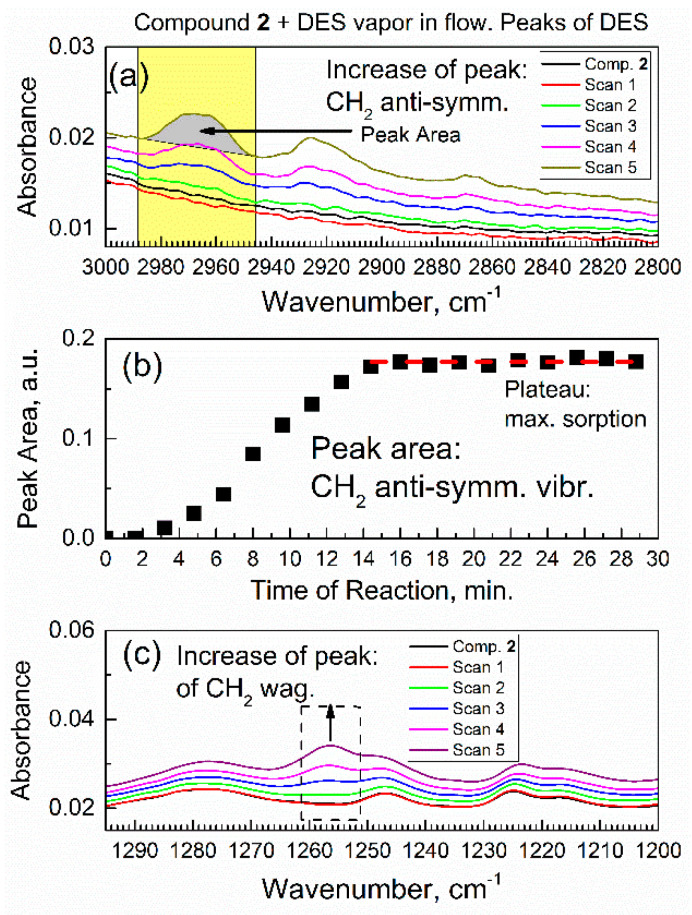
Temporal evolution of in situ ATR-FTIR peaks of adsorbed DES molecules. (**a**) The C–H vibration peaks of DES adsorbate, and peaks of the antisymmetric CH_2_ stretch vibration in the first 9.6 min. (**b**) Time progression of the integrated areas of antisymmetric CH_2_ stretch vibration of DES adsorbate within 30 min. (**c**) The growth of peak due to the C–H wagging vibration of DES adsorbate in the first 9.6 min.

Figure 7a shows how peaks due to the C–H stretch vibrations of adsorbed DES molecules grew during the first six spectral scans (at sorption time of 0–9.6 min). The most intense peak with the maximum at 2965 cm^−1^ (its area is highlighted in yellow) was due to the antisymmetric stretch vibration of the CH_2_ group. The peaks were integrated for each scan; for example, for scan 5, the integrated area is filled in gray. The quantitative details are in Figure 7b, which shows the time progression of peak area of the CH_2_ stretch vibration of the DES molecule within 0–30 min. The integrated IR peak area reflected the total number of CH_2_ groups in the DES molecule (total amount of adsorbed DES). In Figure 7b, in the first 15 min, there was a progressive increase of the peak area, and, subsequently, a plateau was achieved. This illustrated the kinetics of sorption and reaching of a dynamic equilibrium between compound **2** and the vapor of DES (Equation (1))

[Al-MOF-TCPPH_2_] (s) + x DES (v) → [Al-MOF-TCPPH_2_](DES)_x_ (s) (1)

Until reaching equilibrium, the stoichiometric index x was changing. When DES vapor was passed over compound **2** for >30 min, the in situ ATR-FTIR spectra and peak area did not change. Figure 7c shows an increase of the peak area due to functional groups in the DES adsorbate: the C–H bonds underwent the wagging vibrations. The growth of this peak occurred simultaneously with the growth of peak due to the antisymmetric CH_2_ stretch vibration (in Figure 7a) in the DES molecule, as expected. However, the IR absorbance of peak in Figure 7a was much higher, which made that peak much more suitable for kinetic analysis below.

Kinetics of sorption (in liquid phase) have often been investigated using time-dependent ATR-FTIR spectroscopy. Tofan-Lazar et al. [34] reported kinetic ATR-FTIR studies of phosphate adsorption on iron (oxyhydr)oxides by plotting IR absorbance of the product (at 1041 cm^−1^) versus time of reaction. Recently, Kazarian’s group has been active in this field, where one can mention work by Possenti et al. [35] on kinetics of crystallization on marble in aqueous phase, which has been determined via integrated IR absorbance vs. time. In the work of Tofan-Lazar et al. [34], kinetic analysis with numeric curve fitting relies on the Langmuir adsorption kinetics model. The Langmuir adsorption kinetics model for sorption in solution has been used commonly, and the formulas for its pseudo-first- and pseudo-second-order rate law were obtained [36,37]. In kinetic studies of sorption in materials systems of “solid–gas”, the Langmuir adsorption kinetics model has also been used. Brancher et al. [38] reported analytical and numerical solutions for kinetics of sorption in the flow of a polyatomic gas on adsorbing–desorbing surfaces. The in situ time-dependent ATR-FTIR spectroscopy is suitable for studies of sorption and desorption in gas phase (see, e.g., sorption of CO_2_ on organic films [39]).

Recently, we reported a new modality of in situ time-dependent ATR-FTIR spectroscopy in the controlled gaseous environment, which allowed facile mechanistic studies of sorption and desorption of vapor by the powder of sorbent on the ATR crystal [25]. There are no studies of kinetics of sorption of any organosulfur compound on any MOF, to our knowledge, using in situ time-dependent ATR-FTIR spectroscopy in a controlled atmosphere.

**Figure 8:**
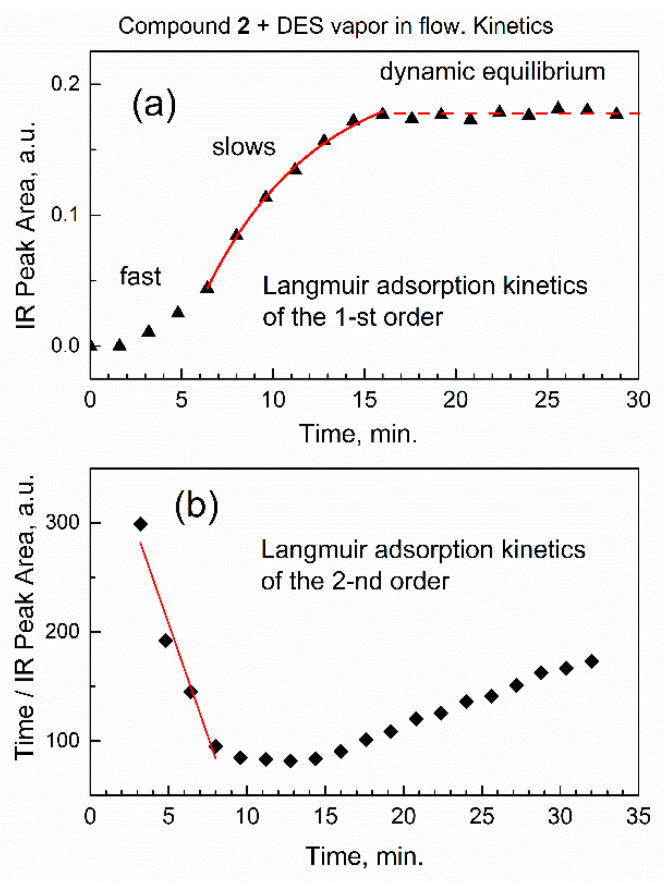
Kinetic analysis of DES sorption based on integrated IR absorbance (peak area) of antisymmetric CH_2_ stretch vibration at 2965 cm^−1^. (**a**) Timescale of the first-order rate law; (**b**) timescale of the second-order rate law.

Figure 8 shows kinetic analysis of the integrated IR absorbance (peak area) versus time for the characteristic vibration of DES adsorbate, the antisymmetric CH_2_ stretch.

In Figure 8a, the kinetic curve has a typical S-shape with an inflection point at ca. 8 min, which indicates that adsorption initially proceeded at the faster rate, and then slowed down. This indicates the action of two distinct kinetic rate laws: the one at the initial time of sorption, and the other at a longer time. First, the similar S-shape of the kinetic curve was reported using ATR-FTIR spectra [34] for sorption in solution. Second, it was shown by theoretical analysis [37] that Langmuir adsorption kinetics can “switch” between the pseudo-first- and the pseudo-second-order rate law: “at high initial concentration of solute (sorbate) the general equation converts to a pseudo-first-order model and at lower initial concentration of solute it converts to a pseudo-second-order model” [37].

The concentration of DES at the adsorption sites of sorbent compound **2** was quantified with regard to the number of available sites in the sorbent. At short times of sorption, there were many sites in the sorbent, and this would correspond to pseudo-second-order rate law, as per ref. [37]. In sorption, this was “the pseudo-second-order model, based on the assumption that the rate-limiting step may be chemical sorption or chemisorption” [40]. This is also consistent with our data, suggesting that at initial sorption time (low coverage by the adsorbate) the chemisorption would prevail over physisorption. At the longer time, limited number of sorption sites remain, and the pseudo-first-order rate law [37] would apply. To process the data in Figure 8a, the equation of Langmuir adsorption kinetics of the pseudo-first-order rate law [34] was used:

A_ν_(*t*) = b’(1 − exp(-r_obs_ *t*)) (2)

where A_ν_(*t*) is the integrated IR absorbance (area) at peak with center at ν cm^−1^, b’ is empirical constant, r_obs_ is observed (effective) kinetic rate constant, and *t* is time of sorption. However, importantly, in Figure 8a, kinetic analysis had to be started at a time other than zero, and the starting integrated IR absorbance A_ν_(*offset*) was also higher than zero. To correct for this, we added a constant to obtain kinetic Equation (3):

A_ν_(*t*) = A_ν_(*offset*) + b’(1 − exp(-r_obs_ *t*)) (3)

In Figure 8a, we fitted the kinetic curve at longer time interval (6.4–16 min, starting at the inflection point) with Equation (3). The kinetic curve in Figure 8a was successfully fitted with an excellent value of the adjusted goodness-of-fit parameter R^2^_adj_ = 0.997. The observed Langmuir adsorption kinetic constant of the pseudo-first-order rate law is r_obs_ = 0.165 ± 0.017 min^−1^.

For the pseudo-second-order rate law, the formula of Langmuir adsorption kinetics in its linearized form is provided in ref. [37]:

*t*/q = 1/*k*_2_(q_e_)^2^ + *t* (1/q_e_) (4)

where q is the current adsorbed amount, q_e_ is the adsorbed amount at equilibrium, *k*_2_ is the pseudo-second-order rate constant of sorption, and *t* is time of sorption. In our model, the adsorbed amount of DES is proportional to peak area A_ν_(*t*), so the formula becomes

*t*/A_ν_(*t*) = 1/*k*_2_(q_e_)^2^ + *t* (1/q_e_) (5)

In Figure 8b, numeric fitting of the initial part of kinetic curve (3–8 min, before the inflection point) with Equation (5) results in parameter R^2^_adj_ = 0.93. The lower quality of fitting, compared to the pseudo-first-order rate law in Figure 8a, is due to a very fast initial reaction rate when it is difficult to promptly collect many FTIR spectra while preserving their quality.

### 3.4. Dynamics and Molecular Mechanism of Interaction of DES with Specific Groups in Compound ***2***

**Figure 9:**
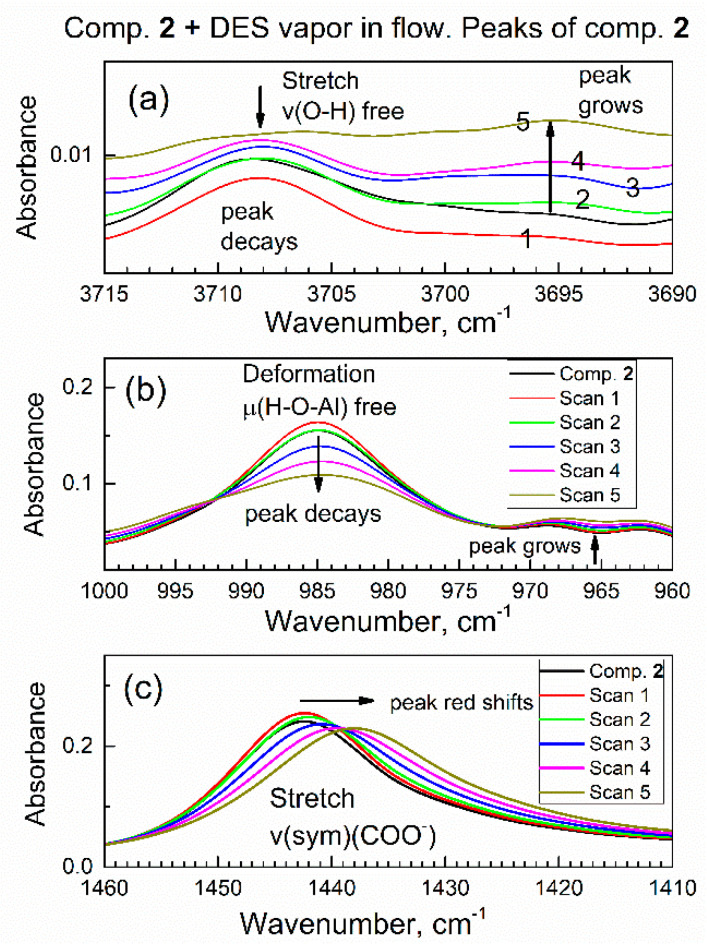
Time evolution of vibrational peaks due to specific functional groups in the in situ ATR-FTIR spectra in the flow of DES vapor. (**a**) Stretching vibration of the O–H group; (**b**) deformation vibration of the O–H group; (**c**) symmetric stretch vibration of the COO^-^ group.

It is of interest to interpret changes in spectra of sorbent compound **2** during sorption of DES vapor. Figure 9 shows the time evolution of selected ATR-FTIR spectroscopic ranges of compound **2**, which correspond to its specific functional groups, during sorption of vapor of DES in the initial period (0–9.6 min). In Figure 9a, the IR peak due to the stretching vibration of free O–H group in compound **2** at ca. 3708 cm^−1^ is progressively decaying, while a new peak is growing at ca. 3695 cm^−1^. In Figure 9a, the numbering 1, 2, 3, 4, 5 means Scan 1, Scan 2, Scan 3, Scan 4, Scan 5. This indicates that the polar O–H bond in compound **2** interacted with polar bonds in DES adsorbate. The same can also be interpreted as “red shift” of the peak due to the O–H group, from 3708 cm^−1^ to 3695 cm^−1^. Spectral red shift (the decrease in the wavenumber) is explained by a harmonic oscillator model: namely, when the adsorbate molecule binds to the given sorption site, the mass of sorption site increases; hence, the wavenumber (energy) of the corresponding vibration decreases.

In Figure 9b, the IR peak due to the deformation vibration of free (nonbonded) H–O–(Al) group at ca. 985 cm^−1^ is progressively decaying. This was expected, since peaks in Figure 9b and Figure 9a belong to the same O–H group in sorbent compound **2**. This confirms that the initially free O–H group interacts with the adsorbate molecule. In the control experiment, a drop of liquid DES was added to the powder of compound **2** on the ATR crystal, and similar shifts of both peaks were promptly observed.

A suitable model to explain this intramolecular interaction is hydrogen bonding between the O–H group in compound **2** and the lone electron pairs of the sulfur atom in the DES molecule.

Similar interactions have been reported for adsorption of organosulfur compounds. First, Glass et al. [41] reported the similar mode of sorption for dimethyl sulfide on alumina Al_2_O_3_ that had surface hydroxy groups H–O–(Al). Second, Wakita et al. [42] reported the similar mechanism of bonding t-butylmercaptan on zeolite with H–O–(Al) groups. Compound **2** is aluminum MOF with essentially the same group, which, in the literature of MOFs, is commonly denoted [27] as μ(O–H).

**Figure 10:**
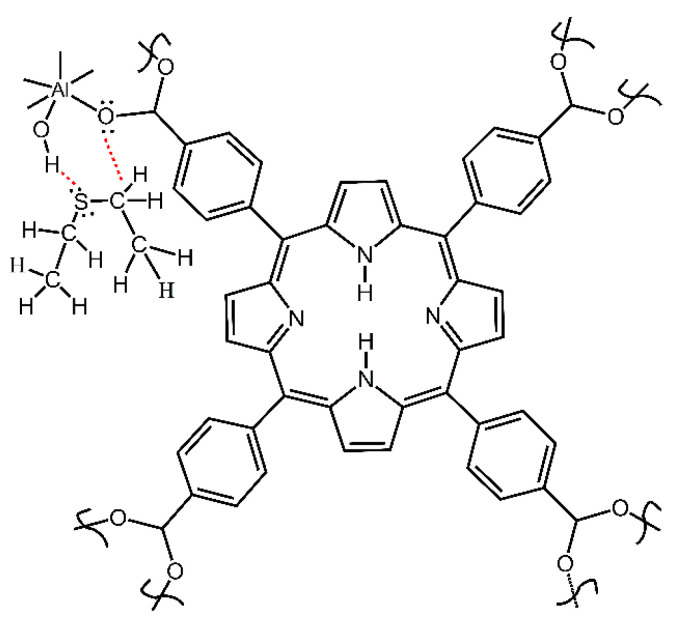
Scheme of bonding of DES molecule to O–H and carboxylate groups in compound **2**.

Further, Figure 9c shows that the IR peak of compound **2** at 1442 cm^−1^ (due to carboxylate COO^-^ group) is gradually shifted to 1437 cm^−1^. The magnitude of this red shift at 5 cm^−1^ exceeds the resolution of the FTIR spectrometer at 4 cm^−1^ and a clear trend is observed. This indicates that the COO^-^ group in compound **2** interacts with the DES adsorbate. The bonding of the DES molecule to the COO^-^ group is also consistent with its bonding to the O–H group, since both groups are connected to an Al atom and are close to each other (Figure 10).

These results indicate that the O–H group in compound **2** underwent the interaction with the DES molecule. The progressive changes of in situ ATR-FTIR spectra reflect progressive sorption of DES by Equation (1). Below, a dynamic mechanistic study of in situ DES sorption is complemented by a ex situ study of sorption and desorption in static conditions.

### 3.5. Static Sorption of DES Vapor by Compound ***2***

Static sorption of DES vapor was conducted in the vapor sorption chamber (see Materials and Methods). The obtained sample, denoted compound **3**, was removed and promptly weighed; there was a significant increase of mass due to the sorption of DES. The reactant compound **2** (aka *act*Al-MOF-TCPPH_2_) has the Hill formula C_48_H_28_O_10_N_4_Al_2_, and the amount of adsorbed DES was determined by gravimetric analysis. On the molar basis, it corresponded to the following equation:

2 Al-MOF-TCPPH_2_ (s) + 7 DES (vap) → [Al-MOF-TCPPH_2_]_2_(DES)_7_ (s) (6)

Compound **2** is a mesoporous MOF with a large nanocavity [43] of orthorhombic space group (Cmmm) with lattice parameters a = 31.978(3) Å, b = 6.5812(4) Å, c = 16.862(2) Å. The molecule of DES has an approximate largest length of <7 Å (by ChemDraw 3D program). This is consistent with few DES molecules per mesopore of adsorption complex compound **3**.

After removal from the vapor sorption chamber, ATR-FTIR spectra of compound **3** were promptly collected, and peaks in the spectra were the same as in the product of the interaction of compound **2** with DES in flowing air. In Figure S7, the characteristic peaks are shown for compound **2** and compound **3**. As expected, the C–H peaks of the DES adsorbate were present only for compound **3**, while the μ(O–H) peak of sorbent was present in both compound **2** and compound **3**, but it was much wider and lower in intensity for compound **3**, indicating the interaction between the Al-MOF “host” and DES adsorbate. Once the formula of compound **3** is simplified to the stoichiometric index of unity, it gives [Al-MOF-TCPPH_2_]_1.0_(DES)_3.5_. Interestingly, the adsorption complex with the similar noninteger stoichiometry was found for other Al-MOFs in our prior work [44], where sorption was conducted using a different method (in solution), and the adsorbate was the oxidized organosulfur compound dimethyl sulfoxide (DMSO). Al-MOFs in ref. [44] and in this work have one common unit besides the Al cation: the μ–OH group. Also importantly, in ref. [44], the μ–OH group of Al-MOF was the binding site for the DMSO adsorbate. This indicates that Al-MOFs are promising sorbents for organosulfur compounds such as DES as well as oxidized organosulfur compounds, and the μ–OH group plays a role in the process.

This is the first study, to our knowledge, where the sorption capacity of DES is determined for any MOF. On the mass basis, the adsorbed amount of DES in this work is quite large, and it corresponds to uptake of ca. 36.5 wt.% or 365 mg/g sorbent. For other classes of sorbents than MOFs, there is only one experimental study of sorption of DES vapor. Namely, ref. [19] provided the equilibrium sorption capacity of DES vapor on 15 types of activated carbons, impregnated with varied amounts of Ni(II), Cr(VI), Co(II), Cu(II), or Cd(II). The maximum uptake of DES in our study at 365 mg/g sorbent was only exceeded by three out of the fifteen sorbents in ref. [19], namely, at 440.1, 431.3, and 418.7 mg/g. However, it is important to note that in ref. [19], the modified activated carbons contained heavy and transition metal dopants as binding sites, while the sorbent in this study, compound **2,** does not have them. It is anticipated that, once transition metal cations are added to the structure of Al-MOF compound **2**, e.g., by post-synthetic modification, the sorption capacity to DES would be even higher. Below, experimental mechanistic studies of the sorption of DES are complemented by quantum chemical DFT computations.

### 3.6. Modeling of Interaction of Compound ***2*** with DES by the DFT Computations

The interaction of Al-MOF in this work (compound **2**) with the vapor of DES was modeled at the two levels of structural complexity. First, the cluster interacting with one DES molecule was considered:

Al-TCPPH_2_ + 1 DES (g) → 1 DES@Al-TCPPH_2_ (7)

The Al-TCPPH_2_ model used here is closely based on the MIL-53(Al) model that we reported in a previous publication [31]. Here, we added the OH(-) and the HCO_2_(-) groups to the edges in such a way that the average oxidation state of all Al atoms was +3, and the overall charge on the Al-TCPPH_2_ model cluster was −1. The important feature of this model is that a DES molecule can intercalate between two porphyrin rings. This is consistent with the large orthorhombic nanocavity in Al-MOF compound **2**.

**Figure 11:**
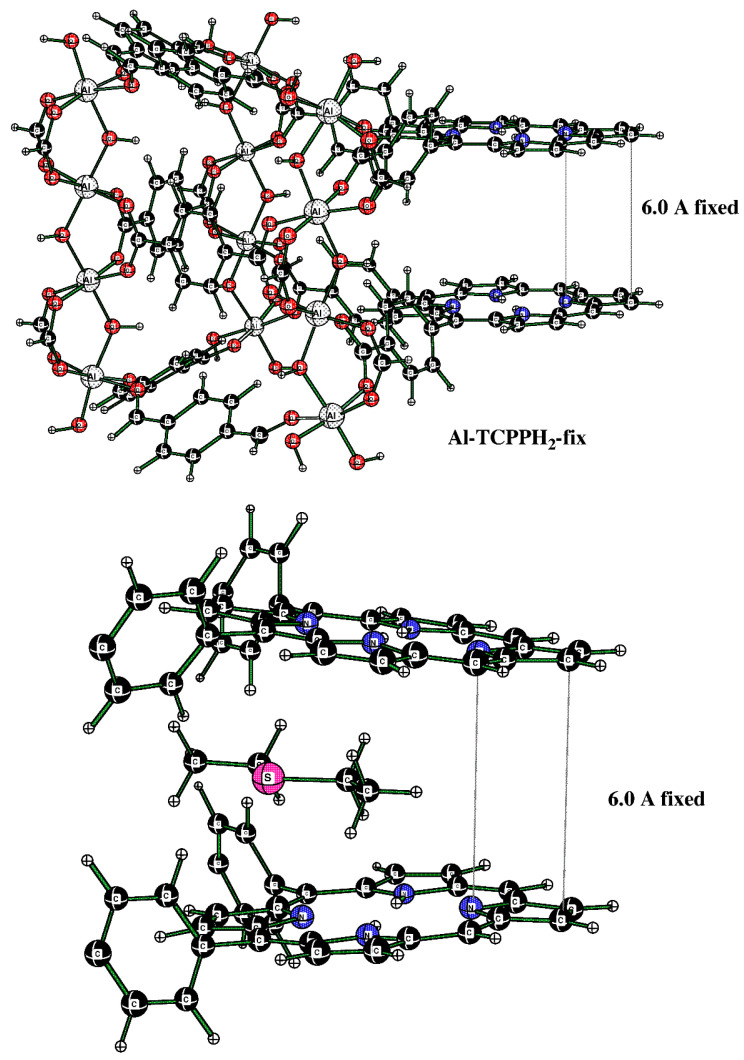
(**Top**) Cluster of the sandwich structure. (**Bottom**) The cutout of the sandwich cluster “DES@Al-TCPPH_2_-fix” showing one DES adsorbate molecule.

Initially, the fully optimized model allowed the two porphyrin rings to approach each other, which is not found in the actual Al-MOF, where the porphyrin rings are separated by about 6.0 Å. While this would not affect the binding of DES molecules to the H–O–Al groups, it would affect the DES molecule in the sandwich (porphyrin–porphyrin) motif. For that reason, we created a new model, Al-TCPPH_2_-fix, where the separation of the top and bottom pyrrole group was fixed to 6.0 Å (see Figure 11 (top)). Namely, in Figure 11 (top), the structure was the modified full model with the two pyrrole rings of the two linkers fixed at a 6.0 Å distance. Then, this cluster interacting with one DES molecule was considered. In Figure 11 (bottom), the structure is the sandwich cluster “DES@Al-TCPPH_2_-fix” with the pyrrole rings fixed at 6.0 Å, where atoms not close to the binding pocket were removed for a better view of the geometry of the DES molecule.

In Figure 11 (bottom), the distances DES(H)--C(porphyrin) are 2.723 Å and 2.738 Å. At the same time, the sum of the van der Waals (vdW) distances for H (1.20 Å) and C (1.70 Å) atoms indicates that some of the distances DES(H)--C(porphyrin) of H bonds are quite significant.

Table 1 provides computed thermodynamic parameters of clusters in this work. The enthalpy of binding ΔH in the sandwich cluster DES@Al-TCPPH_2_-fix (per DES molecule) is –15.5 kcal/mol, calculated on the molar basis. The computed positive Gibbs molar energy, ΔG = 2.0 kcal/mol, indicates that binding the DES molecule in the sandwich cluster is not favorable.

**Figure 12:**
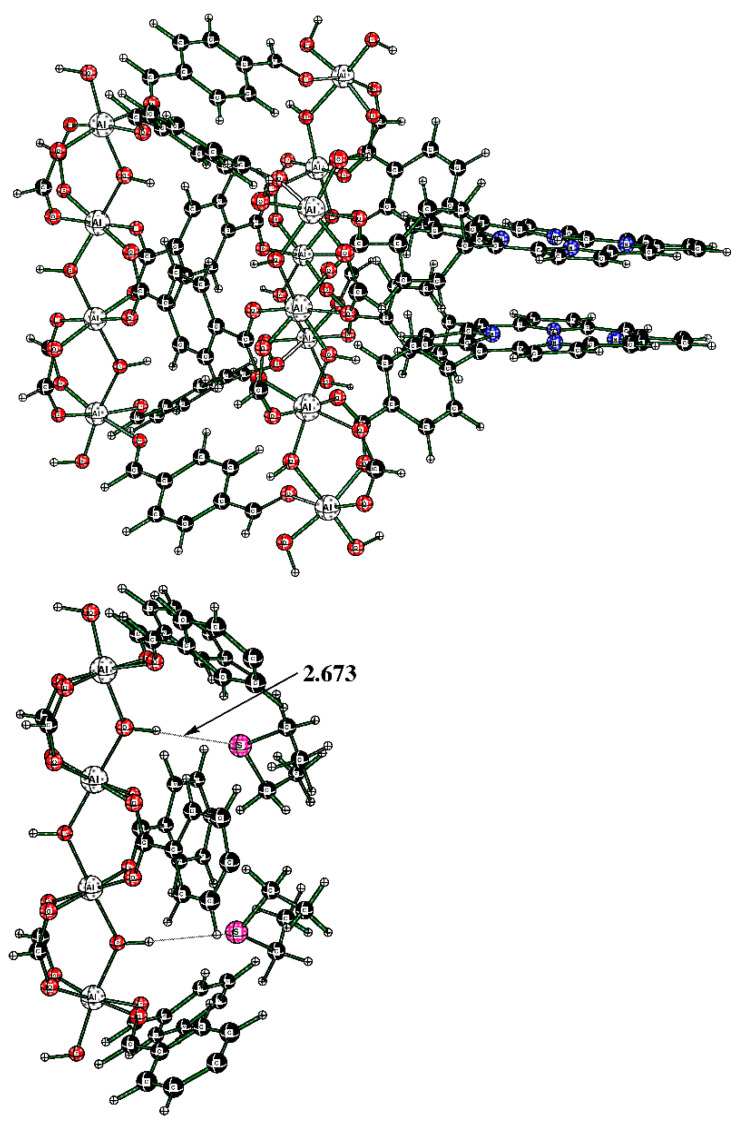
(**Top**) The full cluster Al-TCPPH_2_ without DES molecules. (**Bottom**) The cutout of cluster “2DES@Al-TCPPH_2_” with S---HOAl binding motif, where two DES molecules interact with HOAl sites.

The alternative structure of binding DES to the cluster was also explored; it originates in cluster Al-TCPPH_2_ (without DES molecules) (Figure 12 (top)). To this cluster, two DES molecules were added; to illustrate the binding pocket of DES, we started from the full cluster with the S---HOAl motif and then cut away most of the atoms so that the binding pocket can more clearly be seen (Figure 12 (bottom)).

The cluster in Figure 12 (bottom) features two DES molecules interacting with HOAl sites. The resultant binding enthalpy, when recalculated on a molar basis, was quite significant at ΔH = −19.3 kcal/mol (Table 1). The distance S---HOAl at 2.673 Å was quite short and indicated strong hydrogen bonding. Here, the computed Gibbs molar energy was negative, indicating that binding two DES molecules was favorable. Therefore, the DFT-computed strongest binding occurred in cluster “2DES@Al-TCPPH_2_” at the S---HOAl motif. This finding is consistent with the experiment, where, upon interaction of Al-MOF with DES adsorbate, the shifts of IR spectral peaks were more significant for the O–H group in Al-MOF compound **2** than for the porphyrin rings in the same Al-MOF.

Table S2 shows additional computed thermodynamic parameters, including absolute molar entropy S (cal/mol K). Here, values of S were computed, while for some of the respective clusters there are imaginary frequencies. Next, for clusters which have a nonzero number of imaginary frequencies (NIMAG), the absolute molar entropy S was corrected. Table S3 shows both original (not corrected) and corrected values of S. Two of the structures had imaginary frequencies which did not contribute to the total entropy. It is likely that these imaginary frequencies were artifacts of the calculation. Therefore, we considered the imaginary frequencies as real and added their contribution to the total entropy. In Table S3, the contributions that the imaginary frequencies would have made if they were real are listed as “Correction to Entropy”. Table S4 shows Cartesian coordinates of all clusters in this work.

The NPA charges were calculated for DES@Al-TCPPH_2_-fix (sandwich motif) and 2DES@Al-TCPPH_2_ (S---HOAl motif) at the B3LYP/6-311G(d,p)+D3BJ level. In the sandwich motif, the DES molecule accepts 0.006 electrons from the cage, while in the S---HOAl motif, each DES molecule donates 0.015 electrons to the cage. Thus, the degree of electron transfer in the binding process is quite small.

### 3.7. Static Desorption of DES from Adsorption Complex Compound ***3***, and Regeneration of Sorbent Compound ***2***

**Figure 13:**
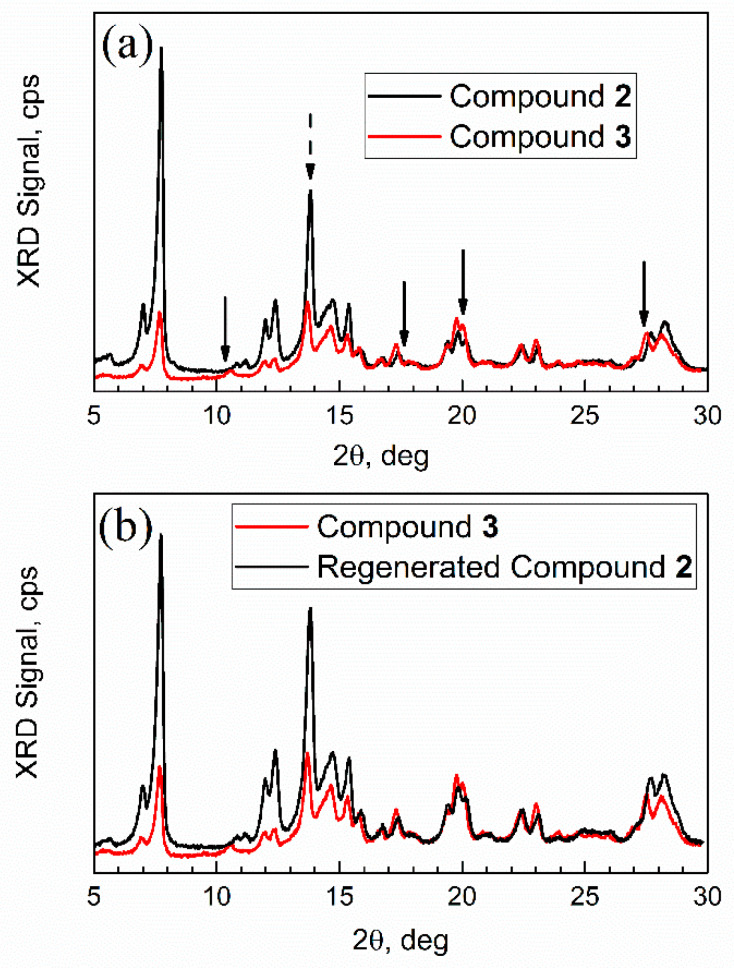
Powder XRD patterns. (**a**) Compound **2** (sorbent) vs. compound **3** (adsorption complex); (**b**) compound **3** vs. regenerated compound **2**.

The freshly prepared adsorption complex compound **3** (on the XRD plate) was promptly transferred from the vapor sorption chamber to the sample compartment of the XRD instrument. Figure 13 shows its XRD pattern versus patterns of activated MOF compound **2**, and regenerated sorbent (obtained in vacuum at 30 °C; see Section 2).

First, the XRD pattern of compound **2** was consistent with that in ref. [43]; however analysis of its nanocrystal size was not previously reported. The numeric analysis of a sharp high intensity peak 2θ = 13.8 degrees in Figure 13a (dashed arrow), which does not overlap with other peaks, was conducted by the Debye–Scherrer equation D = *k* λ/β cos(θ). Here, *k* is a constant, specifically, a shape factor 1.075 for spherical nanoparticles [45], λ is an X-ray wavelength, β is a full-width at the half-maximum (FWHM) of diffraction peak (in radians), and θ is Bragg angle. This analysis gives an average nanocrystal size of compound **2** of 32 nm.

Second, the XRD patterns of mesoporous Al-MOF compound **2** and adsorption complex (aka “spent sorbent”) compound **3** had some differences (marked with arrows) in peaks at ca. 2θ = 10 degrees, 17 degrees, and 27 degrees, but mainly in relative peak intensity. In addition, one new peak (or a significantly shifted one) can be seen in Figure 13a for compound **3** at ca. 11 degrees. Such minor changes are common for MOFs after sorption of “guest” molecules. For example, sorption on mesoporous MOF-5 resulted in the loss of one XRD peak due to the change of orientation in the crystal plane by adsorbate [46]. Third, after regeneration (see Figure 13b), the XRD pattern of compound **3** changed and became very similar to that of compound **2**, as expected. Upon regeneration, the sample had the mass of compound **2**.

## 4. Conclusions

The mechanism of reversible interaction of the porphyrin aluminum metal–organic framework compound **2** with DES was investigated by the complementary in situ and ex-situ experimental methods and DFT computations. An advancement of the method of in situ time-dependent ATR-FTIR spectroscopy in a controlled atmosphere is described, by upgrading it with the newly designed spectroscopic mini-chamber. Under dynamic conditions of sorption (in flow of vapor in air), compound **2** quickly sorbs vapor of DES. In the obtained material, DES adsorbate molecules are weakly bonded to the carboxylate anion and the O–H group. Kinetics of sorption changes when the sorption is proceeding; at the initial time, it follows kinetic pseudo-second-order rate law, which subsequently changes to the pseudo-first-order rate law, and then dynamic equilibrium occurs. For the Langmuir adsorption kinetics of the pseudo-first-order rate law, the rate constant is r_obs_ = 0.165 ± 0.017 min^−1^. Additionally, sorption of DES occurs under static (no flow) conditions in saturated vapor. Herein, compound **2** forms an adsorption complex denoted compound **3**, namely, [Al-MOF-TCPPH_2_]_2_(DES)_7_. This corresponds to a very high adsorbed amount of DES at 36.5 wt.% (365 mg/g sorbent), one of the highest reported. Notably, the adsorption complex compound **3** was facilely regenerated in a vacuum by gentle heating at 30 °C. The DFT quantum chemical computations using a small cluster model suggest that a binding site at O–H group is more favorable than at the porphyrin group of linker in compound **2**. The presented advanced material, porphyrin aluminum metal–organic framework compound **2**, has significant potential for removal of DES and related VOSCs from air. The described mini-apparatus and methodology of in situ time-dependent ATR-FTIR spectroscopy in a controlled atmosphere can be utilized in research of sorption, desorption, work with hazardous materials, separations, heterogeneous catalysis, photocatalysis, chemosensing, and other applications that benefit from controlled atmosphere, in situ spectroscopic reaction monitoring, and studies of kinetics in “solid–gas” materials systems.

## Figures and Tables

**Table 1:** Binding energies (ΔH and ΔG) of DES molecule(s) in Al-TCPPH_2_ clusters (a).

Species	Binding Motif	ΔH (g, 298 K) per DES, kcal/mol (b)	ΔG (g, 298 K) per DES, kcal/mol (b)
DES@Al-TCPPH_2_-fix	Sandwich	−15.5/1 = −15.5	2.0/1 = 2.0
2DES@Al-TCPPH_2_	S---HOAl	−38.6/2 = −19.3	−15.9/2 = −8.0

(a) Results are at the B3LYP/6-311G(d,p)+D3BJ//B3LYP/6-31G(d)+D3BJ level for the binding of one or two DES molecules in Al-TCPPH_2_. A correction for basis set superposition error (BSSE) was made using the counterpoise procedure at the B3LYP/6-311G(d,p)+D3BJ level. Zero-point, heat capacity, and entropy corrections (298 K) were made using unscaled frequencies at the B3LYP/6-31G(d)+D3BJ level. A modified correction for entropy at 298 K was made due to an inconsistent number of imaginary frequencies. See Table S3 for details. (b) Here, “per DES” means that for values of ΔH and ΔG of binding in the 2DES@Al-TCPPH_2_ cluster, these values per cluster need to be divided by two.

## Data Availability

Data are available upon request.

## Supplementary Materials

The supporting information can be downloaded at: https://www.mdpi.com/article/10.3390/nano13222916/s1, Figure S1: The starting template for making spectroscopic mini-chamber; Figure S2: Facile in-flow vapor saturation setup for the flow of DES vapor in dried air; Figure S3: The survey ATR-FTIR spectrum of compound **2** actAl-MOF-TCPPH_2_; Figure S4: Vibrational spectra of compound **2**. (a) ATR-FTIR. (b) Raman; Figure S5: The survey ATR-FTIR spectrum of liquid DES; Table S1: Assignments of major ATR-FTIR peaks of DES; Figure S6: The second set (9.7–19.2 min.) of in-situ time-dependent ATR-FTIR spectra of compound **2** in the flow of DES vapor in dried air. (a) high wavenumbers. (b) the mid-IR. (c) low wavenumbers; Figure S7: The ATR-FTIR spectra of actAl-MOF-TCPPH_2_ (compound **2**) and its adsorption complex with DES (compound **3**). (a) the C-H peaks of adsorbate. (b) the O-H peak of sorbent; Table S2: Absolute Energies, E, computed at B3LYP/6-311G(d,p)+D3BJ level, Basis Set Superposition Errors (BSSE), Zero-point Energies (ZPE), Number of Imaginary Frequencies (NIMAG), Heat Capacity Corrections C_p_, and Entropies S at Optimized B3LYP/6-31G(d)+D3BJ Geometries; Table S3: Number (and Values) of Imaginary Frequencies for Calculated Structures and Corrections to Entropy due to Different Number of Imaginary Frequencies (a); Table S4: Optimized Structures of DES, Al-TCPPH_2_, 2DES@Al-TCPPH_2_, and DES@Al-TCPPH_2_ at the B3LYP/6-31G(d)+D3BJ Level.
