# Supplementary material for: Interaction of a Porphyrin Aluminum Metal–Organic Framework with Volatile Organic Sulfur Compound Diethyl Sulfide Studied via In Situ and Ex Situ Experiments and DFT Computations
Source: Nanomaterials (Basel). 2023 Nov 8;13(22):2916. doi: 10.3390/nano13222916 (PMC10675828; doi:10.3390/nano13222916)
Supplement: Supplementary file 1 [file nanomaterials-13-02916-s001.zip › nanomaterials-2664403-supplementary.pdf]

Supplementary Materials

Interaction of a Porphyrin Aluminum Metal–Organic Framework with Volatile Organic Sulfur Compound Diethyl Sulfide Studied via In Situ and Ex Situ Experiments and DFT Computations

Shaheed Ullah [1], Michael L. McKee [2], and Alexander Samokhvalov [1]*

[1] Department of Chemistry, Morgan State University, 1700 East Cold Spring Lane, Baltimore, MD 21251, USA.

[2] Department of Chemistry and Biochemistry, 179 Chemistry Building, Auburn University, Auburn, AL 36849, USA.

* The corresponding author.

Materials and Methods

Instrumental characterization of samples

The FTIR spectra were collected by Nicolet iS10 spectrometer in the ATR-FTIR mode. This spectrometer was equipped with ATR assembly of model Golden Gate (part number GS10500 from Specac, Fort Washington, PA, USA) with a diamond ATR crystal. The software for data acquisition was OMNIC, where spectral resolution was 4 cm^{-1} and optical aperture at "Open".

To avoid effects of water vapor in ambient air on spectra, the interior of the FTIR spectrometer has been purged with the IR purge gas (dried air of very low humidity) at flow rate 30 scfh (standard cubic feet per hour) as measured by flowmeter (model RMA-7 from Dwyer Instruments, Michigan City, IN, USA). Dried air was generated by FT-IR Purge Gas Generator (model 74-5041 Parker Balston, from Parker Hannifin Corporation, Haverhill, MA, USA) which contains a built-in air compressor. This unit creates dried air of the spectroscopic quality; the remaining water vapor content (per specifications) is equivalent to a dewpoint of -100 °F (-73 °C) which corresponds to relative humidity RH<1%. Additionally, this purge gas generator removes CO_2 from the air to less than 1 ppm. To monitor the quality of FTIR spectra continuously and remove any remaining artifacts due to trace water vapor, in the OMNIC program the "Atmospheric Correction" parameter was enabled, and "Spectral Quality Results" parameter set at "H_2O level" ≥95 %.The obtained ATR-FTIR spectra have been plotted in the absorbance mode. Numeric peak fitting of the ATR-FTIR spectra was conducted by OriginPro 2016 program from OriginLab, Northampton, MA, USA.

The Raman spectra were collected using confocal Raman microscope model XploRA Plus from Horiba Scientific, Piscataway, NJ, USA. The magnification of objective was x50 and the 405 nm laser was used at 1 % of maximum power. A small amount of compound 2 was placed on the microscope glass slide, covered with thin microscope glass cover slide, and Raman spectra of specimen were collected; in work with compound 2 and DES, additionally few drops of DES were added. The Raman spectra were collected in the range 70-2000 cm^{-1}.

Powder X-Ray diffraction (XRD) traces were collected by the diffractometer model MiniFlex from Rigaku, Auburn Hills, MI, USA. It uses X-rays at the Cu K-alpha line of 0.15418 nm and increments of the 2θ angle were at 0.02 degrees.

Fabrication of hemi-spherical gas flow spectroscopic mini-chamber

The hemispherical body of the described spectroscopic mini-chamber was fabricated from a template: transparent acrylic dome (hemisphere) of 1" diameter (from SupremeTech), shown in Figure S1. First, the through hole was drilled at the center of the top of the template, to accommodate the screw of the ATR assembly. Then, the hemisphere template was inverted, its internal volume filled with colorless transparent epoxy mixed with hardener (Doctor Resin & Chemicals, Sarzana (SP), Italy), then stainless steel sapphire anvil (product 10531 from Specac) with its tip up was placed inside the mixture. The mixture was leveled to the edges of the inverted hemisphere, to create the empty internal volume of this spectroscopic mini-chamber at only a few cubic millimeters. The mixture inside the template was allowed to solidify for 24 hours. Then, two holes were drilled through the front and rear sides of spectroscopic mini-chamber and two pieces of soft silicone tubing (of 1/16" ID x 1/8" OD) were inserted to the holes, to create gas inlet port (see 4 in Figure 3) and gas outlet port (see 5 in Figure 3). In this design, gas inlet port and gas outlet port can be interchangeably used. Finally, to ensure gas tightness, a flat adhesive mini O-ring was cut from soft silicone adhesive sheet (hardness 50 Durometer) of 1 mm thick, placed between the mini-chamber and the ATR baseplate, and the ATR bridge assembly was locked.

Results and Discussion

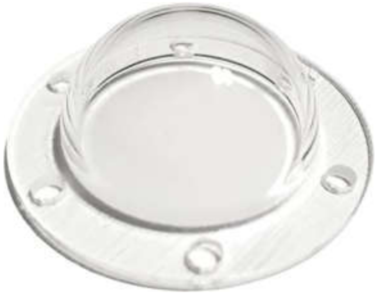

Figure S1. The starting template for making spectroscopic mini-chamber.

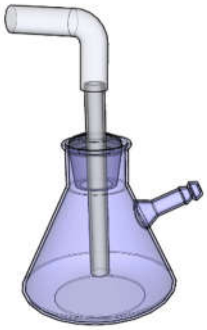

Figure S2. Facile in-flow vapor saturation setup for the flow of DES vapor in dried air.

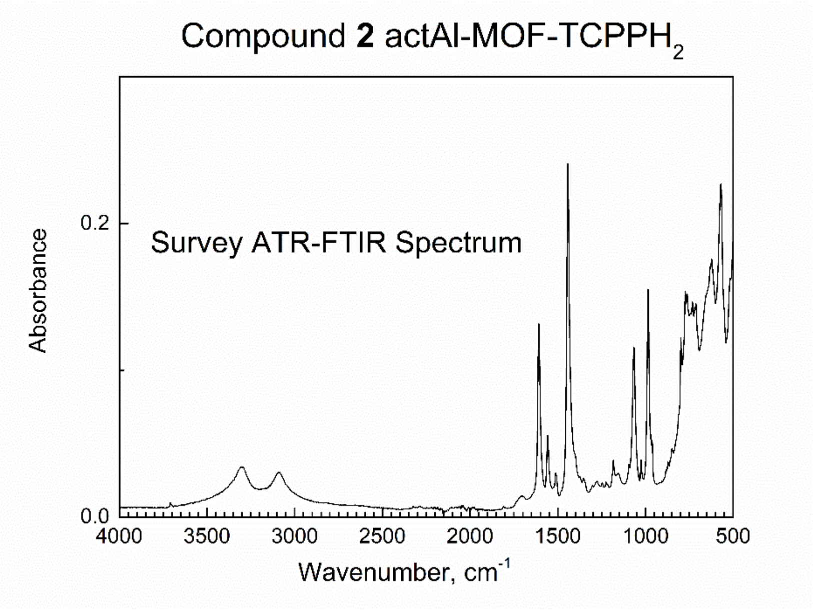

Figure S3. The survey ATR-FTIR spectrum of compound **2** actAl-MOF-TCPPH$_2$.

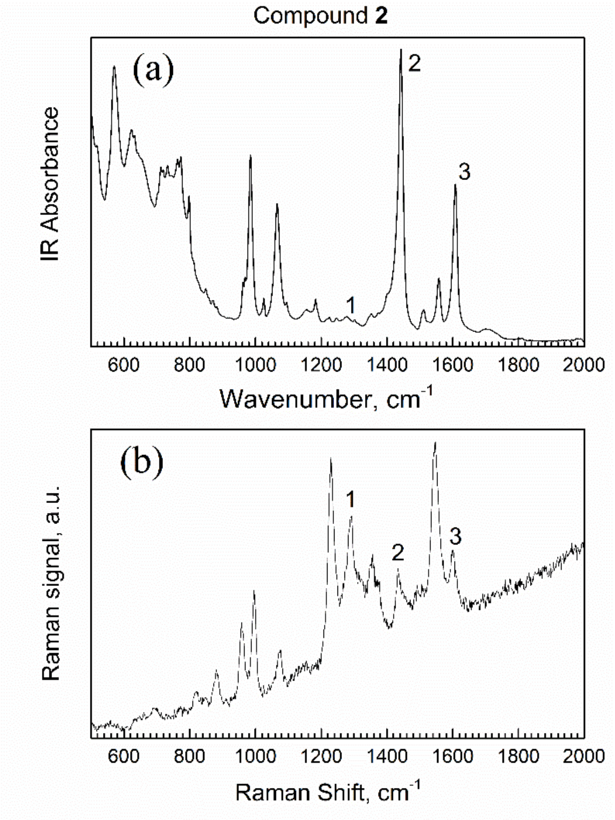

Figure S4. Vibrational spectra of compound **2.** (**a**) ATR-FTIR. (**b**) Raman.

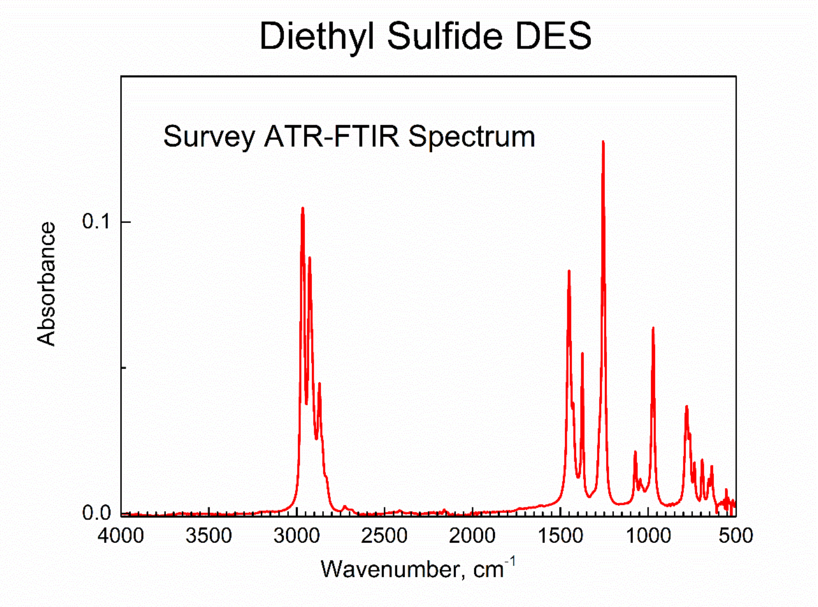

Figure S5. The survey ATR-FTIR spectrum of liquid DES.

Table S1. Assignments of major ATR-FTIR peaks of DES.

The wavenumber (this work), cm^{-1}	The wavenumber (literature), cm^{-1}	Assignments
2965	2970	CH_2 antisymm. str.
2926	2929	CH_3 str, CH_2 sym. str
2870	2871	CH_3 sym. str
1450	1451	CH_3 def
1427	1427	CH_2 sciss
1257	1258	CH_2 wag
1047 w	1046	CH_3 rock
971	972	C-C str.
780 wide	781	-
762 w, sh.	764	-
693	694	S-C str.

Notations: str = stretch; def = deformation; sciss = scission; wag = wagging; rock = rocking.

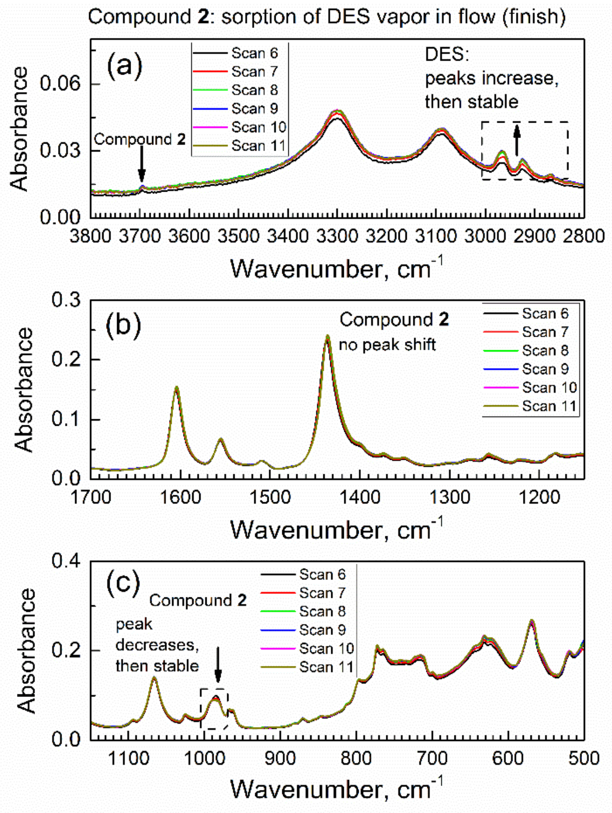

Figure S6. The second set (9.7-19.2 min.) of *in-situ* time-dependent ATR-FTIR spectra of compound **2** in the flow of DES vapor in dried air. (**a**) high wavenumbers. (**b**) the mid-IR. (**c**) low wavenumbers.

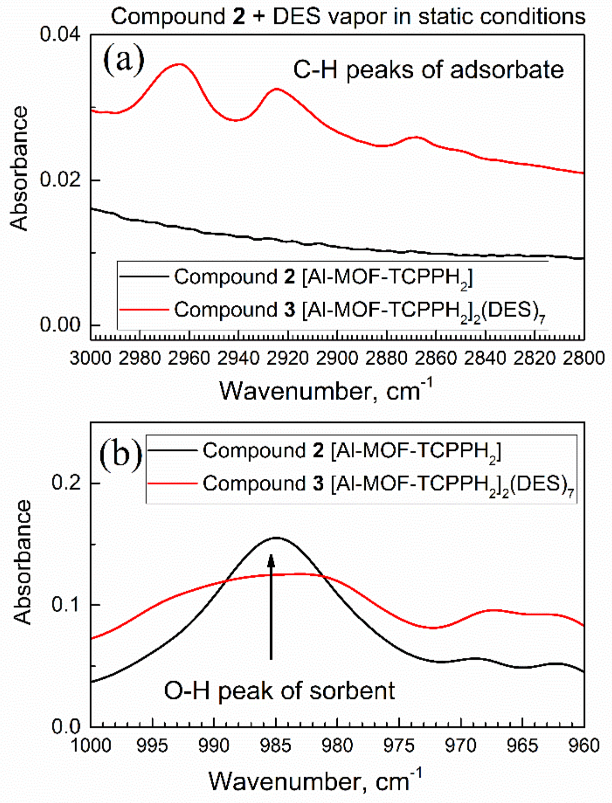

Figure S7. The ATR-FTIR spectra of *act*Al-MOF-TCPPH$_2$ (compound **2**) and its adsorption complex with DES (compound **3**). (**a**) the C-H peaks of adsorbate. (**b**) the O-H peak of sorbent.

Table S2. Absolute Energies, E, computed at B3LYP/6-311G(d,p)+D3BJ level, Basis Set Superposition Errors (BSSE), Zero-point Energies (ZPE), Number of Imaginary Frequencies (NIMAG), Heat Capacity Corrections C_p, and Entropies S at Optimized B3LYP/6-31G(d)+D3BJ Geometries.

Species	PG	Notation	E, Hartree	BSSE, kcal/mol	ZPE, kcal/mol (NIMAG)	C_p, kcal/mol	S, cal/mol-K
DES	C_{2v}	er11	-556.735628	-	84.21(0)	5.28	83.40
Model #1 porphyrin rings close							
Al-TCPPH_2	C_2	er5aa	-12955.778926	-	1378.44(1)	122.82	896.62
2DES@Al-TCPPH_2	C_2	er7a	-14069.323937	5.34	1548.98(2)	133.55	980.13
Model #2 porphyrin rings separated by 6.0 Å							
Al-TCPPH_2-fix	C_s	er5aa-f	-12955.757621	-	1377.37(2)	122.95	908.64
DES@Al-TCPPH_2-fix	C_s	er7bb-f2x	-13512.531138	5.03	1463.82(0)	129.24	953.54

Table S3. Number (and Values) of Imaginary Frequencies for Calculated Structures and Corrections to Entropy due to Different Number of Imaginary Frequencies (a).

Species	Notation	NIMAG (values)	S, cal/mol-K	Correction to Entropy, cal/mol-K	Corrected S, cal/mol-K	-TΔS, kcal/mol (b)
DES	er11	0	83.40	-	83.40	-
	Model #1 porphyrin rings close					
Al-TCPPH_2	er5aa	1 (170i)	896.62	2.44	899.06	-
2DES@Al-TCPPH_2	er7a	2 (170i, 33i)	980.13	8.08	988.21	+23.15
	Model #2 porphyrin rings separated by 6.0 Å					
Al-TCPPH_2-fix	er5aa-f	2 (7i, 6i)	908.64	17.75	926.39	-
DES@Al-TCPPH_2-fix	er7bb-f2x	0	953.54	-	953.54	+16.77

(a) The entropy corrections are very sensitive to small magnitude vibrations. To compute the free energies of binding, imaginary frequencies were considered as real modes and added to the total entropy.

(b) The total TΔS (at 298 K) value in kcal/mol for binding of one or two DES molecules inside Al-TCPPH_2.

Table S4. Optimized Structures of DES, Al-TCPPH$_2$, 2DES@Al-TCPPH$_2$, and DES@Al-TCPPH$_2$ at the B3LYP/6-31G(d)+D3BJ Level.

1. DES aka al-linker11

Standard orientation:

Center Number	Atomic Number	Atomic Type	Coordinates (Angstroms) X	Y	Z
1	16	0	-0.000000	0.000000	0.565368
2	6	0	-0.000000	1.398952	-0.622444
3	1	0	-0.886329	1.319023	-1.262240
4	1	0	0.886329	1.319023	-1.262240
5	6	0	-0.000000	-1.398952	-0.622444
6	1	0	-0.886329	-1.319023	-1.262240
7	1	0	0.886329	-1.319023	-1.262240
8	6	0	0.000000	-2.727524	0.130226
9	1	0	-0.886349	-2.820246	0.766348
10	1	0	0.000000	-3.563675	-0.577854
11	1	0	0.886349	-2.820246	0.766348
12	6	0	0.000000	2.727524	0.130226
13	1	0	0.000000	3.563675	-0.577854
14	1	0	-0.886349	2.820246	0.766348
15	1	0	0.886349	2.820246	0.766348

Model#1 porphyrin rings close

2. Al-TCPPH$_2$ C_s symmetry "Al-TCPPH$_2$" aka al-linker5aa

Standard orientation:

Center Number	Atomic Number	Atomic Type	Coordinates (Angstroms) X	Y	Z
1	8	0	8.506767	6.554381	-0.930434
2	8	0	8.483926	3.392936	0.991094
3	8	0	7.360955	4.213998	-1.415476
4	8	0	9.804561	1.137896	1.479627
5	8	0	6.719184	5.344177	0.972731
6	8	0	9.521708	2.001783	-1.125650
7	8	0	9.556244	5.634728	1.352138
8	8	0	6.748740	2.208534	-0.600563
9	8	0	10.090317	4.199061	-1.123905
10	8	0	7.072264	1.131143	1.785180
11	1	0	9.184908	3.534135	1.646964
12	1	0	6.099648	4.804092	-3.517803
13	1	0	4.321118	4.500025	-5.242739
14	1	0	4.479587	4.607141	1.767403
15	1	0	-2.894191	1.042382	-3.152638
16	1	0	2.894191	1.042382	-3.152638
17	1	0	4.726195	1.306391	-1.474804
18	1	0	5.277623	2.138920	3.076584
19	1	0	-4.321118	4.500025	-5.242739
20	13	0	8.287081	1.679652	0.374016

21	13	0	8.654231	4.998511	-0.083750
22	6	0	5.399452	3.978296	-3.452794
23	6	0	4.404999	3.805215	-4.412515
24	6	0	4.498151	5.186284	2.684565
25	6	0	-3.602408	1.858834	-3.233397
26	6	0	3.602408	1.858834	-3.233397
27	6	0	4.611673	2.010200	-2.291048
28	6	0	4.817292	1.211579	3.399133
29	6	0	-4.404999	3.805215	-4.412515
30	6	0	5.518739	3.072637	-2.393787
31	6	0	3.494806	2.740404	-4.318441
32	6	0	5.669261	5.896539	3.004052
33	6	0	-3.494806	2.740404	-4.318441
34	6	0	6.624643	3.190234	-1.395039
35	6	0	10.256570	0.000000	1.779175
36	6	0	6.833924	5.817188	2.094361
37	6	0	10.211408	3.002444	-1.478007
38	8	0	-8.506767	6.554381	-0.930434
39	8	0	-8.483926	3.392936	0.991094
40	8	0	-10.090317	4.199061	-1.123905
41	8	0	-7.072264	1.131143	1.785180
42	8	0	-9.556244	5.634728	1.352138
43	8	0	-6.748740	2.208534	-0.600563
44	8	0	-6.719184	5.344177	0.972731
45	8	0	-9.521708	2.001783	-1.125650
46	8	0	-7.360955	4.213998	-1.415476
47	8	0	-9.804561	1.137896	1.479627
48	1	0	-9.184908	3.534135	1.646964
49	1	0	-5.277623	2.138920	3.076584
50	1	0	-4.726195	1.306391	-1.474804
51	1	0	-4.479587	4.607141	1.767403
52	1	0	-6.099648	4.804092	-3.517803
53	13	0	-8.287081	1.679652	0.374016
54	13	0	-8.654231	4.998511	-0.083750
55	6	0	-4.817292	1.211579	3.399133
56	6	0	-4.611673	2.010200	-2.291048
57	6	0	-4.498151	5.186284	2.684565
58	6	0	-5.399452	3.978296	-3.452794
59	6	0	-5.396570	-0.000000	2.999753
60	6	0	-5.518739	3.072637	-2.393787
61	6	0	-10.211408	3.002444	-1.478007
62	6	0	-6.614829	-0.000000	2.116493
63	6	0	-6.624643	3.190234	-1.395039
64	8	0	8.160099	0.000000	-0.393894
65	8	0	8.483926	-3.392936	0.991094
66	8	0	6.748740	-2.208534	-0.600563
67	8	0	9.556244	-5.634728	1.352138
68	8	0	7.072264	-1.131143	1.785180
69	8	0	10.090317	-4.199061	-1.123905
70	8	0	9.804561	-1.137896	1.479627
71	8	0	7.360955	-4.213998	-1.415476
72	8	0	9.521708	-2.001783	-1.125650
73	8	0	6.719184	-5.344177	0.972731
74	1	0	8.648787	0.000000	-1.229801
75	1	0	9.184908	-3.534135	1.646964
76	1	0	4.726195	-1.306391	-1.474804

77	1	0	2.894191	-1.042382	-3.152638
78	1	0	5.277623	-2.138920	3.076584
79	1	0	-4.321118	-4.500025	-5.242739
80	1	0	4.321118	-4.500025	-5.242739
81	1	0	6.099648	-4.804092	-3.517803
82	1	0	4.479587	-4.607141	1.767403
83	1	0	-2.894191	-1.042382	-3.152638
84	13	0	8.654231	-4.998511	-0.083750
85	13	0	8.287081	-1.679652	0.374016
86	6	0	4.611673	-2.010200	-2.291048
87	6	0	3.602408	-1.858834	-3.233397
88	6	0	4.817292	-1.211579	3.399133
89	6	0	-4.404999	-3.805215	-4.412515
90	6	0	4.404999	-3.805215	-4.412515
91	6	0	5.399452	-3.978296	-3.452794
92	6	0	4.498151	-5.186284	2.684565
93	6	0	-3.602408	-1.858834	-3.233397
94	6	0	5.518739	-3.072637	-2.393787
95	6	0	3.494806	-2.740404	-4.318441
96	6	0	5.396570	-0.000000	2.999753
97	6	0	-3.494806	-2.740404	-4.318441
98	6	0	6.624643	-3.190234	-1.395039
99	6	0	6.614829	-0.000000	2.116493
100	6	0	10.211408	-3.002444	-1.478007
101	8	0	-8.160099	0.000000	-0.393894
102	8	0	-8.483926	-3.392936	0.991094
103	8	0	-9.521708	-2.001783	-1.125650
104	8	0	-6.719184	-5.344177	0.972731
105	8	0	-9.804561	-1.137896	1.479627
106	8	0	-7.360955	-4.213998	-1.415476
107	8	0	-7.072264	-1.131143	1.785180
108	8	0	-10.090317	-4.199061	-1.123905
109	8	0	-6.748740	-2.208534	-0.600563
110	8	0	-9.556244	-5.634728	1.352138
111	1	0	-8.648787	0.000000	-1.229801
112	1	0	-9.184908	-3.534135	1.646964
113	1	0	-4.479587	-4.607141	1.767403
114	1	0	-6.099648	-4.804092	-3.517803
115	1	0	-5.277623	-2.138920	3.076584
116	1	0	-4.726195	-1.306391	-1.474804
117	13	0	-8.654231	-4.998511	-0.083750
118	13	0	-8.287081	-1.679652	0.374016
119	6	0	-4.498151	-5.186284	2.684565
120	6	0	-5.399452	-3.978296	-3.452794
121	6	0	-4.817292	-1.211579	3.399133
122	6	0	-4.611673	-2.010200	-2.291048
123	6	0	-5.669261	-5.896539	3.004052
124	6	0	-5.518739	-3.072637	-2.393787
125	6	0	-10.211408	-3.002444	-1.478007
126	6	0	-6.833924	-5.817188	2.094361
127	6	0	-10.256570	0.000000	1.779175
128	6	0	-6.624643	-3.190234	-1.395039
129	8	0	0.000000	3.300658	6.163025
130	8	0	0.000000	6.717016	7.354777
131	8	0	-1.384975	1.125278	5.777340
132	8	0	1.412904	4.501571	8.116232

133	8	0	-1.355364	2.237302	8.253193
134	8	0	1.354737	5.344222	5.459428
135	8	0	1.355364	2.237302	8.253193
136	8	0	-1.354737	5.344222	5.459428
137	8	0	1.384975	1.125278	5.777340
138	8	0	-1.412904	4.501571	8.116232
139	1	0	0.844063	3.257528	5.696274
140	1	0	-3.218045	2.144716	4.506653
141	1	0	2.505554	4.679347	3.331254
142	1	0	-2.505554	4.679347	3.331254
143	1	0	3.218045	2.144716	4.506653
144	13	0	0.000000	5.008265	6.900689
145	13	0	0.000000	1.645912	6.988807
146	6	0	-3.672909	1.212446	4.189464
147	6	0	3.415829	5.229334	3.543062
148	6	0	-3.415829	5.229334	3.543062
149	6	0	3.672909	1.212446	4.189464
150	6	0	-3.488805	6.002884	4.720079
151	6	0	3.091320	0.000000	4.589961
152	6	0	-2.372550	6.025450	5.645967
153	6	0	1.815045	3.374094	8.524369
154	6	0	-1.815045	3.374094	8.524369
155	6	0	1.870776	0.000000	5.442552
156	8	0	-0.000000	-3.300658	6.163025
157	8	0	-0.000000	-0.000000	7.829007
158	8	0	-1.354737	-5.344222	5.459428
159	8	0	1.355364	-2.237302	8.253193
160	8	0	-1.412904	-4.501571	8.116232
161	8	0	1.384975	-1.125278	5.777340
162	8	0	1.412904	-4.501571	8.116232
163	8	0	-1.384975	-1.125278	5.777340
164	8	0	1.354737	-5.344222	5.459428
165	8	0	-1.355364	-2.237302	8.253193
166	1	0	-0.844063	-3.257528	5.696274
167	1	0	-0.000000	-0.000000	8.793795
168	1	0	-2.505554	-4.679347	3.331254
169	1	0	3.218045	-2.144716	4.506653
170	1	0	-3.218045	-2.144716	4.506653
171	1	0	2.505554	-4.679347	3.331254
172	13	0	-0.000000	-1.645912	6.988807
173	13	0	-0.000000	-5.008265	6.900689
174	6	0	-3.415829	-5.229334	3.543062
175	6	0	3.672909	-1.212446	4.189464
176	6	0	-3.672909	-1.212446	4.189464
177	6	0	3.415829	-5.229334	3.543062
178	6	0	-3.091320	0.000000	4.589961
179	6	0	3.488805	-6.002884	4.720079
180	6	0	-1.870776	0.000000	5.442552
181	6	0	1.815045	-3.374094	8.524369
182	6	0	-1.815045	-3.374094	8.524369
183	6	0	2.372550	-6.025450	5.645967
184	6	0	5.751098	6.651540	4.179656
185	6	0	3.488805	6.002884	4.720079
186	6	0	5.669261	-5.896539	3.004052
187	6	0	4.655224	-6.718754	5.032577
188	6	0	4.655224	6.718754	5.032577

189	6	0	5.751098	-6.651540	4.179656
190	1	0	6.669622	7.182731	4.411671
191	1	0	4.697338	7.307471	5.945003
192	1	0	6.669622	-7.182731	4.411671
193	1	0	4.697338	-7.307471	5.945003
194	6	0	2.372550	6.025450	5.645967
195	6	0	6.833924	-5.817188	2.094361
196	1	0	2.431068	6.648882	6.547444
197	1	0	7.830778	-6.150232	2.426650
198	6	0	-4.655224	6.718754	5.032577
199	6	0	-5.669261	5.896539	3.004052
200	6	0	-3.488805	-6.002884	4.720079
201	6	0	-5.751098	-6.651540	4.179656
202	1	0	-4.697338	7.307471	5.945003
203	1	0	-6.669622	-7.182731	4.411671
204	6	0	-5.751098	6.651540	4.179656
205	6	0	-4.655224	-6.718754	5.032577
206	1	0	-4.697338	-7.307471	5.945003
207	1	0	-6.669622	7.182731	4.411671
208	6	0	-2.372550	-6.025450	5.645967
209	6	0	-6.833924	5.817188	2.094361
210	1	0	-2.431068	-6.648882	6.547444
211	1	0	-7.830778	6.150232	2.426650
212	1	0	-2.431068	6.648882	6.547444
213	1	0	-7.830778	-6.150232	2.426650
214	1	0	2.431068	-6.648882	6.547444
215	1	0	7.830778	6.150232	2.426650
216	1	0	11.171009	0.000000	2.392652
217	1	0	-11.020494	-2.788846	-2.194598
218	1	0	-11.020494	2.788846	-2.194598
219	1	0	11.020494	2.788846	-2.194598
220	1	0	11.020494	-2.788846	-2.194598
221	1	0	2.673615	3.395889	9.211031
222	1	0	2.673615	-3.395889	9.211031
223	1	0	-2.673615	-3.395889	9.211031
224	1	0	-2.673615	3.395889	9.211031
225	1	0	-11.171009	0.000000	2.392652
226	8	0	-0.000000	-6.717016	7.354777
227	8	0	-8.506767	-6.554381	-0.930434
228	8	0	8.506767	-6.554381	-0.930434
229	1	0	-9.962334	-6.471969	1.087898
230	1	0	-9.962334	6.471969	1.087898
231	1	0	9.962334	-6.471969	1.087898
232	1	0	9.962334	6.471969	1.087898
233	1	0	0.000000	6.852824	8.310315
234	1	0	-7.991528	6.472262	-1.742353
235	1	0	7.991528	6.472262	-1.742353
236	1	0	7.991528	-6.472262	-1.742353
237	1	0	-0.000000	-6.852824	8.310315
238	1	0	-7.991528	-6.472262	-1.742353
239	7	0	2.105482	2.094209	-7.766519
240	7	0	0.000000	2.417405	-5.767940
241	7	0	-2.105482	2.094209	-7.766519
242	7	0	0.000000	1.867820	-9.787028
243	6	0	2.877595	1.876362	-8.881502
244	6	0	4.239143	1.846033	-8.439053

245	6	0	4.252263	2.062281	-7.084895
246	6	0	2.893976	2.239514	-6.650717
247	6	0	2.443077	2.529375	-5.355208
248	6	0	1.090111	2.634723	-4.969760
249	6	0	0.677826	3.028553	-3.624883
250	6	0	-0.677826	3.028553	-3.624883
251	6	0	-1.090111	2.634723	-4.969760
252	6	0	-2.443077	2.529375	-5.355208
253	6	0	-2.893976	2.239514	-6.650717
254	6	0	-4.252263	2.062281	-7.084895
255	6	0	-4.239143	1.846033	-8.439053
256	6	0	-2.877595	1.876362	-8.881502
257	6	0	-2.413835	1.764464	-10.188100
258	6	0	-1.084631	1.793556	-10.609469
259	6	0	-0.677981	1.707809	-12.009403
260	6	0	0.677981	1.707809	-12.009403
261	6	0	1.084631	1.793556	-10.609469
262	6	0	2.413835	1.764464	-10.188100
263	1	0	5.086869	1.677284	-9.089236
264	1	0	5.109304	2.094833	-6.429618
265	1	0	1.342977	3.285682	-2.813639
266	1	0	-1.342977	3.285682	-2.813639
267	1	0	-5.109304	2.094833	-6.429618
268	1	0	-5.086869	1.677284	-9.089236
269	1	0	-3.175099	1.643539	-10.952880
270	1	0	-1.352601	1.636295	-12.853541
271	1	0	1.352601	1.636295	-12.853541
272	1	0	3.175099	1.643539	-10.952880
273	7	0	-2.105482	-2.094209	-7.766519
274	7	0	-0.000000	-2.417405	-5.767940
275	7	0	2.105482	-2.094209	-7.766519
276	7	0	-0.000000	-1.867820	-9.787028
277	6	0	-2.877595	-1.876362	-8.881502
278	6	0	-4.239143	-1.846033	-8.439053
279	6	0	-4.252263	-2.062281	-7.084895
280	6	0	-2.893976	-2.239514	-6.650717
281	6	0	-2.443077	-2.529375	-5.355208
282	6	0	-1.090111	-2.634723	-4.969760
283	6	0	-0.677826	-3.028553	-3.624883
284	6	0	0.677826	-3.028553	-3.624883
285	6	0	1.090111	-2.634723	-4.969760
286	6	0	2.443077	-2.529375	-5.355208
287	6	0	2.893976	-2.239514	-6.650717
288	6	0	4.252263	-2.062281	-7.084895
289	6	0	4.239143	-1.846033	-8.439053
290	6	0	2.877595	-1.876362	-8.881502
291	6	0	2.413835	-1.764464	-10.188100
292	6	0	1.084631	-1.793556	-10.609469
293	6	0	0.677981	-1.707809	-12.009403
294	6	0	-0.677981	-1.707809	-12.009403
295	6	0	-1.084631	-1.793556	-10.609469
296	6	0	-2.413835	-1.764464	-10.188100
297	1	0	-5.086869	-1.677284	-9.089236
298	1	0	-5.109304	-2.094833	-6.429618
299	1	0	-1.342977	-3.285682	-2.813639
300	1	0	1.342977	-3.285682	-2.813639

301	1	0	5.109304	-2.094833	-6.429618
302	1	0	5.086869	-1.677284	-9.089236
303	1	0	3.175099	-1.643539	-10.952880
304	1	0	1.352601	-1.636295	-12.853541
305	1	0	-1.352601	-1.636295	-12.853541
306	1	0	-3.175099	-1.643539	-10.952880
307	1	0	-1.090924	-2.132283	-7.765750
308	1	0	1.090924	-2.132283	-7.765750
309	1	0	-1.090924	2.132283	-7.765750
310	1	0	1.090924	2.132283	-7.765750

----

3. 2DES@Al-TCPPH$_2$ H---HOAl C_2 symmetry "2DES@Al-TCPPH$_2$-S---HOAl" aka al-linker7a

Standard orientation:

Center Number	Atomic Number	Atomic Type	Coordinates (Angstroms) X	Y	Z
1	8	0	6.998292	-7.100733	-1.690319
2	8	0	4.141303	-7.839354	0.539176
3	8	0	4.435351	-6.382164	-1.764332
4	8	0	2.277013	-9.631478	1.112981
5	8	0	5.742550	-5.767380	0.480413
6	8	0	2.733775	-8.948022	-1.534115
7	8	0	6.572303	-8.494432	0.566426
8	8	0	2.440678	-6.230318	-0.731725
9	8	0	4.982619	-9.078973	-1.801431
10	8	0	1.765835	-6.992860	1.680125
11	1	0	4.477839	-8.545284	1.113566
12	1	0	4.592973	-4.931107	-3.731647
13	1	0	3.895906	-3.084666	-5.248494
14	1	0	4.771963	-3.715738	1.457764
15	1	0	0.247563	4.305754	-3.698273
16	1	0	0.602885	-2.182042	-2.650175
17	1	0	1.282790	-4.072380	-1.163884
18	1	0	2.598240	-5.027284	2.793786
19	1	0	4.334791	4.877097	-4.861159
20	13	0	2.365193	-7.948112	0.112299
21	13	0	5.618773	-7.611210	-0.691805
22	6	0	3.724340	-4.323807	-3.504030
23	6	0	3.330826	-3.293055	-4.345828
24	6	0	5.449727	-3.670765	2.303454
25	6	0	1.199753	4.798786	-3.550839
26	6	0	1.487399	-2.770079	-2.877640
27	6	0	1.865959	-3.824912	-2.044789
28	6	0	1.649722	-4.695136	3.200803
29	6	0	3.498895	5.132982	-4.216542
30	6	0	2.974250	-4.620767	-2.362166
31	6	0	2.193609	-2.515299	-4.062695
32	6	0	6.370367	-4.718987	2.483645
33	6	0	2.277013	4.463107	-4.379783
34	6	0	3.323107	-5.828890	-1.550975
35	6	0	1.296065	-10.277155	1.568502
36	6	0	6.374764	-5.842336	1.525067
37	6	0	3.797483	-9.397356	-2.053324
38	8	0	5.703435	9.715774	-0.604514

39	8	0	2.489339	9.193820	1.217183
40	8	0	3.124631	10.885652	-0.860096
41	8	0	0.456522	7.347813	1.896058
42	8	0	4.561479	10.529812	1.668078
43	8	0	1.817882	7.220860	-0.329324
44	8	0	4.650766	7.719620	1.152610
45	8	0	1.085740	9.899448	-1.006157
46	8	0	3.599928	8.231959	-1.246211
47	8	0	-0.064597	10.017027	1.481850
48	1	0	2.486148	9.941230	1.835289
49	1	0	1.610131	5.658640	3.236199
50	1	0	0.523501	5.989167	-1.881768
51	1	0	4.033305	5.384752	1.799154
52	1	0	4.592571	6.610166	-3.075747
53	13	0	0.897460	8.637199	0.511000
54	13	0	4.118866	9.578100	0.193523
55	6	0	0.735899	5.053549	3.448952
56	6	0	1.350174	5.734913	-2.537195
57	6	0	4.602827	5.389580	2.723020
58	6	0	3.649034	6.092768	-3.216649
59	6	0	-0.505940	5.464846	2.948869
60	6	0	2.584307	6.375482	-2.356420
61	6	0	1.950840	10.780376	-1.287305
62	6	0	-0.610991	6.704277	2.106617
63	6	0	2.699088	7.352223	-1.235053
64	8	0	0.613801	-8.125412	-0.450756
65	8	0	-2.489339	-9.193820	1.217183
66	8	0	-1.817882	-7.220860	-0.329324
67	8	0	-4.561479	-10.529812	1.668078
68	8	0	-0.456522	-7.347813	1.896058
69	8	0	-3.124631	-10.885652	-0.860096
70	8	0	0.064597	-10.017027	1.481850
71	8	0	-3.599928	-8.231959	-1.246211
72	8	0	-1.085740	-9.899448	-1.006157
73	8	0	-4.650766	-7.719620	1.152610
74	1	0	0.612818	-8.604933	-1.292446
75	1	0	-2.486148	-9.941230	1.835289
76	1	0	-0.523501	-5.989167	-1.881768
77	1	0	-0.247563	-4.305754	-3.698273
78	1	0	-1.610131	-5.658640	3.236199
79	1	0	-3.895906	3.084666	-5.248494
80	1	0	-4.334791	-4.877097	-4.861159
81	1	0	-4.592571	-6.610166	-3.075747
82	1	0	-4.033305	-5.384752	1.799154
83	1	0	-0.602885	2.182042	-2.650175
84	13	0	-4.118866	-9.578100	0.193523
85	13	0	-0.897460	-8.637199	0.511000
86	6	0	-1.350174	-5.734913	-2.537195
87	6	0	-1.199753	-4.798786	-3.550839
88	6	0	-0.735899	-5.053549	3.448952
89	6	0	-3.330826	3.293055	-4.345828
90	6	0	-3.498895	-5.132982	-4.216542
91	6	0	-3.649034	-6.092768	-3.216649
92	6	0	-4.602827	-5.389580	2.723020
93	6	0	-1.487399	2.770079	-2.877640
94	6	0	-2.584307	-6.375482	-2.356420

95	6	0	-2.277013	-4.463107	-4.379783
96	6	0	0.505940	-5.464846	2.948869
97	6	0	-2.193609	2.515299	-4.062695
98	6	0	-2.699088	-7.352223	-1.235053
99	6	0	0.610991	-6.704277	2.106617
100	6	0	-1.950840	-10.780376	-1.287305
101	8	0	-0.613801	8.125412	-0.450756
102	8	0	-4.141303	7.839354	0.539176
103	8	0	-2.733775	8.948022	-1.534115
104	8	0	-5.742550	5.767380	0.480413
105	8	0	-2.277013	9.631478	1.112981
106	8	0	-4.435351	6.382164	-1.764332
107	8	0	-1.765835	6.992860	1.680125
108	8	0	-4.982619	9.078973	-1.801431
109	8	0	-2.440678	6.230318	-0.731725
110	8	0	-6.572303	8.494432	0.566426
111	1	0	-0.612818	8.604933	-1.292446
112	1	0	-4.477839	8.545284	1.113566
113	1	0	-4.771963	3.715738	1.457764
114	1	0	-4.592973	4.931107	-3.731647
115	1	0	-2.598240	5.027284	2.793786
116	1	0	-1.282790	4.072380	-1.163884
117	13	0	-5.618773	7.611210	-0.691805
118	13	0	-2.365193	7.948112	0.112299
119	6	0	-5.449727	3.670765	2.303454
120	6	0	-3.724340	4.323807	-3.504030
121	6	0	-1.649722	4.695136	3.200803
122	6	0	-1.865959	3.824912	-2.044789
123	6	0	-6.370367	4.718987	2.483645
124	6	0	-2.974250	4.620767	-2.362166
125	6	0	-3.797483	9.397356	-2.053324
126	6	0	-6.374764	5.842336	1.525067
127	6	0	-1.296065	10.277155	1.568502
128	6	0	-3.323107	5.828890	-1.550975
129	8	0	3.255855	0.391283	5.911399
130	8	0	6.653978	0.813917	7.070318
131	8	0	0.963696	1.488072	5.575381
132	8	0	4.663988	-0.919758	7.774559
133	8	0	2.052583	1.597966	8.054437
134	8	0	5.461081	-0.600568	5.119170
135	8	0	2.416346	-1.085628	8.030091
136	8	0	5.073101	2.078611	5.276103
137	8	0	1.276690	-1.288428	5.601671
138	8	0	4.300733	1.878897	7.936335
139	1	0	3.122506	0.603038	4.972957
140	1	0	1.789553	3.549680	4.582969
141	1	0	4.707037	-1.814778	3.112709
142	1	0	4.284749	3.310890	3.203857
143	1	0	2.433633	-2.931912	4.173194
144	13	0	4.950478	0.610784	6.626584
145	13	0	1.640959	0.177419	6.779426
146	6	0	0.833308	3.886065	4.198083
147	6	0	5.419559	-2.626341	3.206611
148	6	0	4.744422	4.251007	3.496554
149	6	0	1.556363	-3.532865	3.959359
150	6	0	5.504638	4.315553	4.683554

151	6	0	0.313503	-3.121509	4.461173
152	6	0	5.651389	3.145078	5.525506
153	6	0	3.605518	-1.439086	8.229812
154	6	0	3.137774	2.156172	8.348800
155	6	0	0.205639	-1.877627	5.271891
156	8	0	-3.255855	-0.391283	5.911399
157	8	0	0.000000	0.000000	7.622323
158	8	0	-5.461081	0.600568	5.119170
159	8	0	-2.052583	-1.597966	8.054437
160	8	0	-4.663988	0.919758	7.774559
161	8	0	-0.963696	-1.488072	5.575381
162	8	0	-4.300733	-1.878897	7.936335
163	8	0	-1.276690	1.288428	5.601671
164	8	0	-5.073101	-2.078611	5.276103
165	8	0	-2.416346	1.085628	8.030091
166	1	0	-3.122506	-0.603038	4.972957
167	1	0	0.000000	0.000000	8.587070
168	1	0	-4.707037	1.814778	3.112709
169	1	0	-1.789553	-3.549680	4.582969
170	1	0	-2.433633	2.931912	4.173194
171	1	0	-4.284749	-3.310890	3.203857
172	13	0	-1.640959	-0.177419	6.779426
173	13	0	-4.950478	-0.610784	6.626584
174	6	0	-5.419559	2.626341	3.206611
175	6	0	-0.833308	-3.886065	4.198083
176	6	0	-1.556363	3.532865	3.959359
177	6	0	-4.744422	-4.251007	3.496554
178	6	0	-0.313503	3.121509	4.461173
179	6	0	-5.504638	-4.315553	4.683554
180	6	0	-0.205639	1.877627	5.271891
181	6	0	-3.137774	-2.156172	8.348800
182	6	0	-3.605518	1.439086	8.229812
183	6	0	-5.651389	-3.145078	5.525506
184	6	0	7.256258	-4.719033	3.566739
185	6	0	6.325295	-2.613375	4.287049
186	6	0	-5.192097	-6.596867	3.141104
187	6	0	-6.097394	-5.519567	5.096108
188	6	0	7.242894	-3.658499	4.466366
189	6	0	-5.923203	-6.666709	4.332510
190	1	0	7.948976	-5.546189	3.691757
191	1	0	7.928135	-3.635868	5.309351
192	1	0	-6.357072	-7.613357	4.641157
193	1	0	-6.675339	-5.546789	6.015948
194	6	0	6.269312	-1.527335	5.248789
195	6	0	-5.022486	-7.808603	2.314795
196	1	0	6.940267	-1.529149	6.117788
197	1	0	-5.200180	-8.811509	2.735305
198	6	0	6.097394	5.519567	5.096108
199	6	0	5.192097	6.596867	3.141104
200	6	0	-6.325295	2.613375	4.287049
201	6	0	-7.256258	4.719033	3.566739
202	1	0	6.675339	5.546789	6.015948
203	1	0	-7.948976	5.546189	3.691757
204	6	0	5.923203	6.666709	4.332510
205	6	0	-7.242894	3.658499	4.466366
206	1	0	-7.928135	3.635868	5.309351

207	1	0	6.357072	7.613357	4.641157
208	6	0	-6.269312	1.527335	5.248789
209	6	0	5.022486	7.808603	2.314795
210	1	0	-6.940267	1.529149	6.117788
211	1	0	5.200180	8.811509	2.735305
212	1	0	6.283252	3.202023	6.421218
213	1	0	-6.918368	6.774743	1.745644
214	1	0	-6.283252	-3.202023	6.421218
215	1	0	6.918368	-6.774743	1.745644
216	1	0	1.546995	-11.194487	2.123278
217	1	0	-3.654591	10.164011	-2.831164
218	1	0	1.625613	11.548974	-2.006692
219	1	0	3.654591	-10.164011	-2.831164
220	1	0	-1.625613	-11.548974	-2.006692
221	1	0	3.745999	-2.305745	8.891768
222	1	0	-3.073367	-2.990651	9.062644
223	1	0	-3.745999	2.305745	8.891768
224	1	0	3.073367	2.990651	9.062644
225	1	0	-1.546995	11.194487	2.123278
226	8	0	-6.653978	-0.813917	7.070318
227	8	0	-6.998292	7.100733	-1.690319
228	8	0	-5.703435	-9.715774	-0.604514
229	1	0	-7.426852	8.725577	0.176851
230	1	0	5.344305	11.051685	1.443228
231	1	0	-5.344305	-11.051685	1.443228
232	1	0	7.426852	-8.725577	0.176851
233	1	0	6.796862	0.786767	8.024420
234	1	0	5.714178	9.268671	-1.459874
235	1	0	6.723066	-6.555142	-2.437170
236	1	0	-5.714178	-9.268671	-1.459874
237	1	0	-6.796862	-0.786767	8.024420
238	1	0	-6.723066	6.555142	-2.437170
239	7	0	1.464437	-1.259522	-7.517217
240	7	0	1.802799	0.920531	-5.628497
241	7	0	2.070174	2.885514	-7.807197
242	7	0	1.765835	0.686572	-9.694101
243	6	0	1.276346	-2.086804	-8.598638
244	6	0	1.146354	-3.416398	-8.082282
245	6	0	1.316754	-3.362276	-6.723218
246	6	0	1.536456	-1.989350	-6.356685
247	6	0	1.802990	-1.487307	-5.072743
248	6	0	1.872305	-0.119597	-4.741566
249	6	0	2.089348	0.371888	-3.383573
250	6	0	2.134719	1.725789	-3.469254
251	6	0	1.979716	2.054753	-4.885733
252	6	0	2.113492	3.368363	-5.380213
253	6	0	2.135679	3.734836	-6.730174
254	6	0	2.232248	5.064930	-7.262496
255	6	0	2.230915	4.974652	-8.631666
256	6	0	2.126560	3.590298	-8.985135
257	6	0	2.086879	3.046782	-10.265094
258	6	0	1.927723	1.699840	-10.593009
259	6	0	1.852643	1.204000	-11.963570
260	6	0	1.619510	-0.130104	-11.869066
261	6	0	1.562272	-0.434637	-10.442071
262	6	0	1.315716	-1.710456	-9.936191

263	1	0	0.943465	-4.288327	-8.687820
264	1	0	1.279028	-4.182376	-6.022863
265	1	0	2.201919	-0.242851	-2.501952
266	1	0	2.295338	2.439985	-2.674765
267	1	0	2.292151	5.956881	-6.656983
268	1	0	2.295531	5.782982	-9.347789
269	1	0	2.174268	3.752991	-11.085542
270	1	0	1.954778	1.809802	-12.855734
271	1	0	1.481006	-0.849744	-12.665409
272	1	0	1.146126	-2.504829	-10.655269
273	7	0	-1.464437	1.259522	-7.517217
274	7	0	-1.802799	-0.920531	-5.628497
275	7	0	-2.070174	-2.885514	-7.807197
276	7	0	-1.765835	-0.686572	-9.694101
277	6	0	-1.276346	2.086804	-8.598638
278	6	0	-1.146354	3.416398	-8.082282
279	6	0	-1.316754	3.362276	-6.723218
280	6	0	-1.536456	1.989350	-6.356685
281	6	0	-1.802990	1.487307	-5.072743
282	6	0	-1.872305	0.119597	-4.741566
283	6	0	-2.089348	-0.371888	-3.383573
284	6	0	-2.134719	-1.725789	-3.469254
285	6	0	-1.979716	-2.054753	-4.885733
286	6	0	-2.113492	-3.368363	-5.380213
287	6	0	-2.135679	-3.734836	-6.730174
288	6	0	-2.232248	-5.064930	-7.262496
289	6	0	-2.230915	-4.974652	-8.631666
290	6	0	-2.126560	-3.590298	-8.985135
291	6	0	-2.086879	-3.046782	-10.265094
292	6	0	-1.927723	-1.699840	-10.593009
293	6	0	-1.852643	-1.204000	-11.963570
294	6	0	-1.619510	0.130104	-11.869066
295	6	0	-1.562272	0.434637	-10.442071
296	6	0	-1.315716	1.710456	-9.936191
297	1	0	-0.943465	4.288327	-8.687820
298	1	0	-1.279028	4.182376	-6.022863
299	1	0	-2.201919	0.242851	-2.501952
300	1	0	-2.295338	-2.439985	-2.674765
301	1	0	-2.292151	-5.956881	-6.656983
302	1	0	-2.295531	-5.782982	-9.347789
303	1	0	-2.174268	-3.752991	-11.085542
304	1	0	-1.954778	-1.809802	-12.855734
305	1	0	-1.481006	0.849744	-12.665409
306	1	0	-1.146126	2.504829	-10.655269
307	1	0	-1.588284	0.253790	-7.569845
308	1	0	-1.954815	-1.878959	-7.747757
309	1	0	1.954815	1.878959	-7.747757
310	1	0	1.588284	-0.253790	-7.569845
311	16	0	2.678117	1.017913	2.364841
312	16	0	-2.678117	-1.017913	2.364841
313	6	0	-1.196051	-1.630096	1.460918
314	1	0	-0.754995	-0.781650	0.930115
315	1	0	-0.487922	-1.925449	2.236987
316	6	0	1.196051	1.630096	1.460918
317	1	0	0.754995	0.781650	0.930115
318	1	0	0.487922	1.925449	2.236987

319	6	0	-3.483047	-0.005332	1.056347
320	1	0	-3.605762	-0.624720	0.161872
321	1	0	-4.488299	0.196211	1.440282
322	6	0	3.483047	0.005332	1.056347
323	1	0	3.605762	0.624720	0.161872
324	1	0	4.488299	-0.196211	1.440282
325	6	0	-2.754613	1.296738	0.725128
326	1	0	-3.308197	1.864773	-0.032090
327	1	0	-2.630008	1.924700	1.613403
328	1	0	-1.757200	1.107492	0.315439
329	6	0	2.754613	-1.296738	0.725128
330	1	0	1.757200	-1.107492	0.315439
331	1	0	3.308197	-1.864773	-0.032090
332	1	0	2.630008	-1.924700	1.613403
333	6	0	-1.477379	-2.791812	0.511993
334	1	0	-1.838568	-3.669112	1.055601
335	1	0	-2.220577	-2.526239	-0.246928
336	1	0	-0.557847	-3.080965	-0.010912
337	6	0	1.477379	2.791812	0.511993
338	1	0	1.838568	3.669112	1.055601
339	1	0	2.220577	2.526239	-0.246928
340	1	0	0.557847	3.080965	-0.010912

Model#2 distal pyrrole rings fixed at 6.0 A separation

4. Al-TCPPH$_2$ C$_s$ symmetry "Al-TCPPH$_2$ sandwich-fix" aka al-linker5aa-fix

Standard orientation:

Center Number	Atomic Number	Atomic Type	Coordinates (Angstroms) X	Y	Z
1	8	0	0.823145	-8.605457	6.577152
2	8	0	-1.024349	-8.405760	3.382696
3	8	0	1.355523	-7.378114	4.285904
4	8	0	-1.543928	-9.682543	1.136589
5	8	0	-1.029171	-6.740683	5.404791
6	8	0	1.068829	-9.517344	1.993966
7	8	0	-1.466968	-9.564802	5.574607
8	8	0	0.684869	-6.740889	2.232824
9	8	0	1.025595	-10.111379	4.183596
10	8	0	-1.728990	-6.936747	1.131638
11	1	0	-1.716178	-9.078403	3.486107
12	1	0	3.166473	-5.953581	5.211040
13	1	0	4.899727	-4.157320	5.128864
14	1	0	-1.838762	-4.535041	4.616815
15	1	0	3.636975	3.101580	1.171764
16	1	0	3.600693	-3.092710	1.182290
17	1	0	1.883902	-4.908648	1.267461
18	1	0	-3.038492	-5.150334	2.138710
19	1	0	4.894134	4.110574	5.146244
20	13	0	-0.370446	-8.215787	1.680236
21	13	0	0.002138	-8.674029	5.002064
22	6	0	3.272924	-5.323635	4.334816
23	6	0	4.237404	-4.321547	4.284166
24	6	0	-2.747540	-4.536889	5.209314
25	6	0	3.531225	3.717441	2.059649

26	6	0	3.500918	-3.717397	2.064697
27	6	0	2.542141	-4.723843	2.108964
28	6	0	-3.363030	-4.692658	1.210757
29	6	0	4.243416	4.289373	4.295458
30	6	0	2.422973	-5.535204	3.244712
31	6	0	4.366346	-3.507440	3.148309
32	6	0	-3.052931	-5.684717	5.962427
33	6	0	4.381100	3.488502	3.151490
34	6	0	1.409756	-6.633903	3.269378
35	6	0	-1.862457	-10.123230	0.000000
36	6	0	-2.143948	-6.850192	5.894109
37	6	0	1.389738	-10.228654	2.989909
38	8	0	0.788289	8.522428	6.612167
39	8	0	-0.972901	8.424636	3.376105
40	8	0	1.069705	10.087785	4.254544
41	8	0	-1.645203	7.004388	1.130388
42	8	0	-1.466157	9.523684	5.581128
43	8	0	0.771187	6.794339	2.210529
44	8	0	-1.056638	6.667440	5.359435
45	8	0	1.134923	9.571581	2.044086
46	8	0	1.366313	7.348242	4.310532
47	8	0	-1.461468	9.746109	1.137359
48	1	0	-1.673853	9.087236	3.481207
49	1	0	-2.994637	5.256609	2.139230
50	1	0	1.936746	4.933649	1.254224
51	1	0	-1.808513	4.373603	4.719649
52	1	0	3.171699	5.917472	5.228315
53	13	0	-0.290834	8.274347	1.680228
54	13	0	0.012482	8.643923	5.018239
55	6	0	-3.336862	4.809953	1.212220
56	6	0	2.581350	4.732524	2.102491
57	6	0	-2.753250	4.421318	5.250836
58	6	0	3.285407	5.297745	4.345786
59	6	0	-2.913028	5.369926	0.000000
60	6	0	2.453554	5.531049	3.246207
61	6	0	1.447257	10.244513	3.069448
62	6	0	-1.989778	6.556319	0.000000
63	6	0	1.451795	6.640833	3.270034
64	8	0	0.401914	-8.106527	0.000000
65	8	0	-1.024349	-8.405760	-3.382696
66	8	0	0.684869	-6.740889	-2.232824
67	8	0	-1.466968	-9.564802	-5.574607
68	8	0	-1.728990	-6.936747	-1.131638
69	8	0	1.025595	-10.111379	-4.183596
70	8	0	-1.543928	-9.682543	-1.136589
71	8	0	1.355523	-7.378114	-4.285904
72	8	0	1.068829	-9.517344	-1.993966
73	8	0	-1.029171	-6.740683	-5.404791
74	1	0	1.218266	-8.627638	0.000000
75	1	0	-1.716178	-9.078403	-3.486107
76	1	0	1.883902	-4.908648	-1.267461
77	1	0	3.600693	-3.092710	-1.182290
78	1	0	-3.038492	-5.150334	-2.138710
79	1	0	4.894134	4.110574	-5.146244
80	1	0	4.899727	-4.157320	-5.128864
81	1	0	3.166473	-5.953581	-5.211040

82	1	0	-1.838762	-4.535041	-4.616815
83	1	0	3.636975	3.101580	-1.171764
84	13	0	0.002138	-8.674029	-5.002064
85	13	0	-0.370446	-8.215787	-1.680236
86	6	0	2.542141	-4.723843	-2.108964
87	6	0	3.500918	-3.717397	-2.064697
88	6	0	-3.363030	-4.692658	-1.210757
89	6	0	4.243416	4.289373	-4.295458
90	6	0	4.237404	-4.321547	-4.284166
91	6	0	3.272924	-5.323635	-4.334816
92	6	0	-2.747540	-4.536889	-5.209314
93	6	0	3.531225	3.717441	-2.059649
94	6	0	2.422973	-5.535204	-3.244712
95	6	0	4.366346	-3.507440	-3.148309
96	6	0	-2.953815	-5.266974	0.000000
97	6	0	4.381100	3.488502	-3.151490
98	6	0	1.409756	-6.633903	-3.269378
99	6	0	-2.061333	-6.480836	0.000000
100	6	0	1.389738	-10.228654	-2.989909
101	8	0	0.488937	8.196908	0.000000
102	8	0	-0.972901	8.424636	-3.376105
103	8	0	1.134923	9.571581	-2.044086
104	8	0	-1.056638	6.667440	-5.359435
105	8	0	-1.461468	9.746109	-1.137359
106	8	0	1.366313	7.348242	-4.310532
107	8	0	-1.645203	7.004388	-1.130388
108	8	0	1.069705	10.087785	-4.254544
109	8	0	0.771187	6.794339	-2.210529
110	8	0	-1.466157	9.523684	-5.581128
111	1	0	1.303381	8.720339	0.000000
112	1	0	-1.673853	9.087236	-3.481207
113	1	0	-1.808513	4.373603	-4.719649
114	1	0	3.171699	5.917472	-5.228315
115	1	0	-2.994637	5.256609	-2.139230
116	1	0	1.936746	4.933649	-1.254224
117	13	0	0.012482	8.643923	-5.018239
118	13	0	-0.290834	8.274347	-1.680228
119	6	0	-2.753250	4.421318	-5.250836
120	6	0	3.285407	5.297745	-4.345786
121	6	0	-3.336862	4.809953	-1.212220
122	6	0	2.581350	4.732524	-2.102491
123	6	0	-3.105240	5.627546	-5.882323
124	6	0	2.453554	5.531049	-3.246207
125	6	0	1.447257	10.244513	-3.069448
126	6	0	-2.193540	6.791304	-5.790580
127	6	0	-1.778346	10.186935	0.000000
128	6	0	1.451795	6.640833	-3.270034
129	8	0	-6.218025	0.045335	3.301408
130	8	0	-7.457112	-0.134538	6.701521
131	8	0	-5.856908	1.484127	1.123785
132	8	0	-8.212296	-1.422948	4.424271
133	8	0	-8.283523	1.431098	2.282381
134	8	0	-5.555085	-1.423088	5.268650
135	8	0	-8.313178	-1.295770	2.160589
136	8	0	-5.539557	1.284751	5.437712
137	8	0	-5.792264	-1.289835	1.126152

138	8	0	-8.170005	1.404173	4.545716
139	1	0	-5.323862	-0.317764	3.248694
140	1	0	-4.513195	3.259928	2.145025
141	1	0	-3.408599	-2.565640	4.645258
142	1	0	-3.379270	2.404178	4.813872
143	1	0	-4.493651	-3.111731	2.144770
144	13	0	-6.982162	-0.034984	5.000763
145	13	0	-7.037901	0.095181	1.641739
146	6	0	-4.177419	3.702346	1.213338
147	6	0	-3.610303	-3.457927	5.227583
148	6	0	-3.614119	3.340918	5.307268
149	6	0	-4.168499	-3.558435	1.211319
150	6	0	-4.828519	3.453156	6.015570
151	6	0	-4.576781	-2.979423	0.000000
152	6	0	-5.762704	2.343014	6.040067
153	6	0	-8.608634	-1.785228	3.281144
154	6	0	-8.563965	1.852266	3.428963
155	6	0	-5.447388	-1.769394	0.000000
156	8	0	-6.218025	0.045335	-3.301408
157	8	0	-7.893912	0.086143	0.000000
158	8	0	-5.539557	1.284751	-5.437712
159	8	0	-8.313178	-1.295770	-2.160589
160	8	0	-8.170005	1.404173	-4.545716
161	8	0	-5.792264	-1.289835	-1.126152
162	8	0	-8.212296	-1.422948	-4.424271
163	8	0	-5.856908	1.484127	-1.123785
164	8	0	-5.555085	-1.423088	-5.268650
165	8	0	-8.283523	1.431098	-2.282381
166	1	0	-5.323862	-0.317764	-3.248694
167	1	0	-8.691862	-0.460689	0.000000
168	1	0	-3.379270	2.404178	-4.813872
169	1	0	-4.493651	-3.111731	-2.144770
170	1	0	-4.513195	3.259928	-2.145025
171	1	0	-3.408599	-2.565640	-4.645258
172	13	0	-7.037901	0.095181	-1.641739
173	13	0	-6.982162	-0.034984	-5.000763
174	6	0	-3.614119	3.340918	-5.307268
175	6	0	-4.168499	-3.558435	-1.211319
176	6	0	-4.177419	3.702346	-1.213338
177	6	0	-3.610303	-3.457927	-5.227583
178	6	0	-4.603195	3.141219	0.000000
179	6	0	-4.774980	-3.509459	-6.021334
180	6	0	-5.503586	1.958180	0.000000
181	6	0	-8.608634	-1.785228	-3.281144
182	6	0	-8.563965	1.852266	-3.428963
183	6	0	-5.705448	-2.398705	-6.019279
184	6	0	-4.214620	-5.745083	6.740210
185	6	0	-4.774980	-3.509459	6.021334
186	6	0	-3.052931	-5.684717	-5.962427
187	6	0	-5.069184	-4.649444	-6.785990
188	6	0	-5.069184	-4.649444	6.785990
189	6	0	-4.214620	-5.745083	-6.740210
190	1	0	-4.434810	-6.646267	7.305027
191	1	0	-5.970514	-4.672741	7.392380
192	1	0	-4.434810	-6.646267	-7.305027
193	1	0	-5.970514	-4.672741	-7.392380

194	6	0	-5.705448	-2.398705	6.019279
195	6	0	-2.143948	-6.850192	-5.894109
196	1	0	-6.576054	-2.411932	6.686933
197	1	0	-2.476635	-7.843349	-6.235958
198	6	0	-5.174603	4.655219	6.652670
199	6	0	-3.105240	5.627546	5.882323
200	6	0	-4.828519	3.453156	-6.015570
201	6	0	-4.316458	5.746164	-6.573607
202	1	0	-6.116375	4.727999	7.190003
203	1	0	-4.572987	6.690668	-7.044649
204	6	0	-4.316458	5.746164	6.573607
205	6	0	-5.174603	4.655219	-6.652670
206	1	0	-6.116375	4.727999	-7.190003
207	1	0	-4.572987	6.690668	7.044649
208	6	0	-5.762704	2.343014	-6.040067
209	6	0	-2.193540	6.791304	5.790580
210	1	0	-6.707447	2.454515	-6.588170
211	1	0	-2.541275	7.796299	6.081749
212	1	0	-6.707447	2.454515	6.588170
213	1	0	-2.541275	7.796299	-6.081749
214	1	0	-6.576054	-2.411932	-6.686933
215	1	0	-2.476635	-7.843349	6.235958
216	1	0	-2.513093	-11.011574	0.000000
217	1	0	2.149203	11.074655	-2.891129
218	1	0	2.149203	11.074655	2.891129
219	1	0	2.085052	-11.055454	2.774253
220	1	0	2.085052	-11.055454	-2.774253
221	1	0	-9.306357	-2.635240	3.265409
222	1	0	-9.306357	-2.635240	-3.265409
223	1	0	-9.243775	2.714786	-3.476173
224	1	0	-9.243775	2.714786	3.476173
225	1	0	-2.426758	11.077031	0.000000
226	8	0	-7.457112	-0.134538	-6.701521
227	8	0	0.788289	8.522428	-6.612167
228	8	0	0.823145	-8.605457	-6.577152
229	1	0	-1.250807	9.932617	-6.430890
230	1	0	-1.250807	9.932617	6.430890
231	1	0	-1.221661	-10.030179	-6.386315
232	1	0	-1.221661	-10.030179	6.386315
233	1	0	-8.412028	-0.208185	6.821103
234	1	0	1.616388	8.028622	6.570936
235	1	0	1.669161	-8.144405	6.522226
236	1	0	1.669161	-8.144405	-6.522226
237	1	0	-8.412028	-0.208185	-6.821103
238	1	0	1.616388	8.028622	-6.570936
239	7	0	7.873615	-2.121460	3.040963
240	7	0	5.852193	-0.012875	3.026855
241	7	0	7.883216	2.090132	3.041685
242	7	0	9.915855	-0.018519	2.999995
243	6	0	9.006667	-2.897703	3.002808
244	6	0	8.570490	-4.261923	2.987237
245	6	0	7.199608	-4.271334	3.015567
246	6	0	6.747100	-2.908056	3.055941
247	6	0	5.421882	-2.453022	3.089002
248	6	0	5.023670	-1.100639	3.081225
249	6	0	3.626413	-0.685265	3.179778

250	6	0	3.629650	0.670307	3.179896
251	6	0	5.028778	1.078861	3.082151
252	6	0	5.432352	2.429308	3.091560
253	6	0	6.758975	2.879816	3.059176
254	6	0	7.215138	4.241982	3.022809
255	6	0	8.585976	4.228912	2.993618
256	6	0	9.018328	2.863399	3.005278
257	6	0	10.328998	2.396665	2.992597
258	6	0	10.744045	1.064414	2.999994
259	6	0	12.146085	0.655252	2.999994
260	6	0	12.143174	-0.701760	2.999994
261	6	0	10.739366	-1.104915	2.999995
262	6	0	10.318924	-2.435463	2.991364
263	1	0	9.236652	-5.113436	2.952246
264	1	0	6.544527	-5.129140	3.003112
265	1	0	2.778117	-1.349896	3.254785
266	1	0	2.784793	1.339403	3.254596
267	1	0	6.562101	5.101412	3.013804
268	1	0	9.254444	5.078706	2.960991
269	1	0	11.103004	3.158148	2.977141
270	1	0	12.994479	1.328538	2.997372
271	1	0	12.988673	-1.378683	2.997375
272	1	0	11.090052	-3.199856	2.975146
273	7	0	7.883216	2.090132	-3.041685
274	7	0	5.852193	-0.012875	-3.026855
275	7	0	7.873615	-2.121460	-3.040963
276	7	0	9.915855	-0.018519	-2.999995
277	6	0	9.018328	2.863399	-3.005278
278	6	0	8.585976	4.228912	-2.993618
279	6	0	7.215138	4.241982	-3.022809
280	6	0	6.758975	2.879816	-3.059176
281	6	0	5.432352	2.429308	-3.091560
282	6	0	5.028778	1.078861	-3.082151
283	6	0	3.629650	0.670307	-3.179896
284	6	0	3.626413	-0.685265	-3.179778
285	6	0	5.023670	-1.100639	-3.081225
286	6	0	5.421882	-2.453022	-3.089002
287	6	0	6.747100	-2.908056	-3.055941
288	6	0	7.199608	-4.271334	-3.015567
289	6	0	8.570490	-4.261923	-2.987237
290	6	0	9.006667	-2.897703	-3.002808
291	6	0	10.318924	-2.435463	-2.991364
292	6	0	10.739366	-1.104915	-2.999995
293	6	0	12.143174	-0.701760	-2.999994
294	6	0	12.146085	0.655252	-2.999994
295	6	0	10.744045	1.064414	-2.999994
296	6	0	10.328998	2.396665	-2.992597
297	1	0	9.254444	5.078706	-2.960991
298	1	0	6.562101	5.101412	-3.013804
299	1	0	2.784793	1.339403	-3.254596
300	1	0	2.778117	-1.349896	-3.254785
301	1	0	6.544527	-5.129140	-3.003112
302	1	0	9.236652	-5.113436	-2.952246
303	1	0	11.090052	-3.199856	-2.975146
304	1	0	12.988673	-1.378683	-2.997375
305	1	0	12.994479	1.328538	-2.997372

306	1	0	11.103004	3.158148	-2.977141
307	1	0	7.875238	1.074948	-3.032882
308	1	0	7.867180	-1.106323	-3.034445
309	1	0	7.875238	1.074948	3.032882
310	1	0	7.867180	-1.106323	3.034445

----

5. DES@Al-TCPPH$_2$ sandwich C_s symmetry "DES@Al-TCPPH$_2$ sandwich-fix" aka al-linker7bb-fix2x

Standard orientation:

Center Number	Atomic Number	Atomic Type	Coordinates (Angstroms) X	Y	Z
1	8	0	-1.314073	8.668629	6.583648
2	8	0	0.513090	8.556093	3.375970
3	8	0	-1.764418	7.369027	4.313278
4	8	0	0.903399	9.860663	1.135719
5	8	0	0.661783	6.928642	5.420537
6	8	0	-1.680766	9.478476	1.994176
7	8	0	0.892793	9.778274	5.546966
8	8	0	-1.068767	6.748450	2.262491
9	8	0	-1.648248	10.118525	4.171232
10	8	0	1.312924	7.135228	1.131729
11	1	0	1.155055	9.279060	3.459028
12	1	0	-3.461791	5.848692	5.279367
13	1	0	-5.010321	3.889004	5.283227
14	1	0	1.615699	4.777803	4.638412
15	1	0	-3.388204	-3.434792	1.442854
16	1	0	-3.518972	2.715867	1.436052
17	1	0	-2.005669	4.702511	1.424883
18	1	0	2.759340	5.459628	2.138768
19	1	0	-4.054961	-4.380174	5.572587
20	13	0	-0.142646	8.300323	1.681586
21	13	0	-0.511437	8.774600	5.001153
22	6	0	-3.487407	5.159170	4.442902
23	6	0	-4.347263	4.064525	4.441269
24	6	0	2.529321	4.849440	5.219033
25	6	0	-3.114168	-4.004130	2.326055
26	6	0	-3.505070	3.401266	2.276837
27	6	0	-2.656510	4.503217	2.269081
28	6	0	3.119279	5.028949	1.210954
29	6	0	-3.501704	-4.543382	4.652175
30	6	0	-2.644929	5.392195	3.351068
31	6	0	-4.371866	3.175128	3.356406
32	6	0	2.763060	6.023921	5.956700
33	6	0	-3.834422	-3.799002	3.512376
34	6	0	-1.756551	6.594056	3.318728
35	6	0	1.184759	10.327050	0.000000
36	6	0	1.773814	7.122321	5.889333
37	6	0	-2.040633	10.182082	2.982571
38	8	0	0.239961	-8.605342	6.642388
39	8	0	1.881604	-8.261156	3.361302
40	8	0	0.050998	-10.140527	4.264266
41	8	0	2.340077	-6.766777	1.130396
42	8	0	2.555666	-9.358696	5.530054
43	8	0	-0.080497	-6.837901	2.249150

44	8	0	1.856275	-6.588965	5.389469
45	8	0	-0.124412	-9.624395	2.058841
46	8	0	-0.499067	-7.441388	4.379546
47	8	0	2.462953	-9.517636	1.136038
48	1	0	2.651888	-8.847524	3.426814
49	1	0	3.540263	-4.915680	2.138941
50	1	0	-1.541128	-5.130322	1.362054
51	1	0	2.364508	-4.232702	4.736755
52	1	0	-2.212422	-6.068296	5.480672
53	13	0	1.138947	-8.180179	1.683399
54	13	0	0.986333	-8.606984	5.027964
55	6	0	3.844901	-4.441825	1.212329
56	6	0	-2.092604	-4.947479	2.277983
57	6	0	3.303842	-4.175754	5.276641
58	6	0	-2.477264	-5.485033	4.605822
59	6	0	3.466323	-5.032813	0.000000
60	6	0	-1.772346	-5.698006	3.416493
61	6	0	-0.340090	-10.328773	3.088764
62	6	0	2.644717	-6.291536	0.000000
63	6	0	-0.698981	-6.736892	3.353109
64	8	0	-0.904678	8.115608	0.000000
65	8	0	0.513090	8.556093	-3.375970
66	8	0	-1.068767	6.748450	-2.262491
67	8	0	0.892793	9.778274	-5.546966
68	8	0	1.312924	7.135228	-1.131729
69	8	0	-1.648248	10.118525	-4.171232
70	8	0	0.903399	9.860663	-1.135719
71	8	0	-1.764418	7.369027	-4.313278
72	8	0	-1.680766	9.478476	-1.994176
73	8	0	0.661783	6.928642	-5.420537
74	1	0	-1.764396	8.561770	0.000000
75	1	0	1.155055	9.279060	-3.459028
76	1	0	-2.005669	4.702511	-1.424883
77	1	0	-3.518972	2.715867	-1.436052
78	1	0	2.759340	5.459628	-2.138768
79	1	0	-4.054961	-4.380174	-5.572587
80	1	0	-5.010321	3.889004	-5.283227
81	1	0	-3.461791	5.848692	-5.279367
82	1	0	1.615699	4.777803	-4.638412
83	1	0	-3.388204	-3.434792	-1.442854
84	13	0	-0.511437	8.774600	-5.001153
85	13	0	-0.142646	8.300323	-1.681586
86	6	0	-2.656510	4.503217	-2.269081
87	6	0	-3.505070	3.401266	-2.276837
88	6	0	3.119279	5.028949	-1.210954
89	6	0	-3.501704	-4.543382	-4.652175
90	6	0	-4.347263	4.064525	-4.441269
91	6	0	-3.487407	5.159170	-4.442902
92	6	0	2.529321	4.849440	-5.219033
93	6	0	-3.114168	-4.004130	-2.326055
94	6	0	-2.644929	5.392195	-3.351068
95	6	0	-4.371866	3.175128	-3.356406
96	6	0	2.666234	5.568763	0.000000
97	6	0	-3.834422	-3.799002	-3.512376
98	6	0	-1.756551	6.594056	-3.318728
99	6	0	1.680479	6.707460	0.000000

100	6	0	-2.040633	10.182082	-2.982571
101	8	0	0.350666	-8.179988	0.000000
102	8	0	1.881604	-8.261156	-3.361302
103	8	0	-0.124412	-9.624395	-2.058841
104	8	0	1.856275	-6.588965	-5.389469
105	8	0	2.462953	-9.517636	-1.136038
106	8	0	-0.499067	-7.441388	-4.379546
107	8	0	2.340077	-6.766777	-1.130396
108	8	0	0.050998	-10.140527	-4.264266
109	8	0	-0.080497	-6.837901	-2.249150
110	8	0	2.555666	-9.358696	-5.530054
111	1	0	-0.406975	-8.782906	0.000000
112	1	0	2.651888	-8.847524	-3.426814
113	1	0	2.364508	-4.232702	-4.736755
114	1	0	-2.212422	-6.068296	-5.480672
115	1	0	3.540263	-4.915680	-2.138941
116	1	0	-1.541128	-5.130322	-1.362054
117	13	0	0.986333	-8.606984	-5.027964
118	13	0	1.138947	-8.180179	-1.683399
119	6	0	3.303842	-4.175754	-5.276641
120	6	0	-2.477264	-5.485033	-4.605822
121	6	0	3.844901	-4.441825	-1.212329
122	6	0	-2.092604	-4.947479	-2.277983
123	6	0	3.775291	-5.332407	-5.923336
124	6	0	-1.772346	-5.698006	-3.416493
125	6	0	-0.340090	-10.328773	-3.088764
126	6	0	2.996816	-6.588176	-5.829724
127	6	0	2.825855	-9.923104	0.000000
128	6	0	-0.698981	-6.736892	-3.353109
129	8	0	6.335670	0.532423	3.301165
130	8	0	7.552822	0.805551	6.702144
131	8	0	6.094417	-0.928660	1.123785
132	8	0	8.201811	2.154996	4.427288
133	8	0	8.508128	-0.683162	2.283592
134	8	0	5.553575	1.940237	5.273220
135	8	0	8.319798	2.036314	2.163931
136	8	0	5.760236	-0.768251	5.436356
137	8	0	5.809639	1.830391	1.126214
138	8	0	8.390557	-0.665309	4.546766
139	1	0	5.411379	0.809427	3.246180
140	1	0	4.896338	-2.804257	2.144997
141	1	0	3.319532	2.923020	4.665432
142	1	0	3.719501	-2.109129	4.820784
143	1	0	4.371311	3.542107	2.144774
144	13	0	7.087079	0.670687	5.000808
145	13	0	7.160473	0.548909	1.642091
146	6	0	4.595321	-3.271458	1.213510
147	6	0	3.465423	3.833220	5.236656
148	6	0	4.046527	-3.010521	5.327463
149	6	0	4.011827	3.962073	1.211512
150	6	0	5.259650	-2.987478	6.046389
151	6	0	4.464354	3.417594	0.000000
152	6	0	6.077148	-1.788591	6.062276
153	6	0	8.571390	2.547897	3.285325
154	6	0	8.820226	-1.080561	3.430483
155	6	0	5.428028	2.280706	0.000000

156	8	0	6.335670	0.532423	-3.301165
157	8	0	8.013570	0.627167	0.000000
158	8	0	5.760236	-0.768251	-5.436356
159	8	0	8.319798	2.036314	-2.163931
160	8	0	8.390557	-0.665309	-4.546766
161	8	0	5.809639	1.830391	-1.126214
162	8	0	8.201811	2.154996	-4.427288
163	8	0	6.094417	-0.928660	-1.123785
164	8	0	5.553575	1.940237	-5.273220
165	8	0	8.508128	-0.683162	-2.283592
166	1	0	5.411379	0.809427	-3.246180
167	1	0	8.766103	1.234943	0.000000
168	1	0	3.719501	-2.109129	-4.820784
169	1	0	4.371311	3.542107	-2.144774
170	1	0	4.896338	-2.804257	-2.144997
171	1	0	3.319532	2.923020	-4.665432
172	13	0	7.160473	0.548909	-1.642091
173	13	0	7.087079	0.670687	-5.000808
174	6	0	4.046527	-3.010521	-5.327463
175	6	0	4.011827	3.962073	-1.211512
176	6	0	4.595321	-3.271458	-1.213510
177	6	0	3.465423	3.833220	-5.236656
178	6	0	4.975113	-2.678673	0.000000
179	6	0	4.632946	3.973933	-6.015185
180	6	0	5.779360	-1.429097	0.000000
181	6	0	8.571390	2.547897	-3.285325
182	6	0	8.820226	-1.080561	-3.430483
183	6	0	5.640145	2.932086	-6.012720
184	6	0	3.927211	6.173202	6.718650
185	6	0	4.632946	3.973933	6.015185
186	6	0	2.763060	6.023921	-5.956700
187	6	0	4.856125	5.139897	-6.764642
188	6	0	4.856125	5.139897	6.764642
189	6	0	3.927211	6.173202	-6.718650
190	1	0	4.091220	7.093556	7.271548
191	1	0	5.760815	5.232233	7.359331
192	1	0	4.091220	7.093556	-7.271548
193	1	0	5.760815	5.232233	-7.359331
194	6	0	5.640145	2.932086	6.012720
195	6	0	1.773814	7.122321	-5.889333
196	1	0	6.514296	3.015556	6.670540
197	1	0	2.041539	8.140620	-6.213807
198	6	0	5.722888	-4.139643	6.701201
199	6	0	3.775291	-5.332407	5.923336
200	6	0	5.259650	-2.987478	-6.046389
201	6	0	4.985406	-5.315721	-6.626533
202	1	0	6.662015	-4.107597	7.247034
203	1	0	5.335979	-6.223317	-7.109341
204	6	0	4.985406	-5.315721	6.626533
205	6	0	5.722888	-4.139643	-6.701201
206	1	0	6.662015	-4.107597	-7.247034
207	1	0	5.335979	-6.223317	7.109341
208	6	0	6.077148	-1.788591	-6.062276
209	6	0	2.996816	-6.588176	5.829724
210	1	0	7.019411	-1.794457	-6.625621
211	1	0	3.449398	-7.549978	6.120680

212	1	0	7.019411	-1.794457	6.625621
213	1	0	3.449398	-7.549978	-6.120680
214	1	0	6.514296	3.015556	-6.670540
215	1	0	2.041539	8.140620	6.213807
216	1	0	1.759269	11.266407	0.000000
217	1	0	-0.960238	-11.224077	-2.922251
218	1	0	-0.960238	-11.224077	2.922251
219	1	0	-2.803366	10.946272	2.763868
220	1	0	-2.803366	10.946272	-2.763868
221	1	0	9.198649	3.451205	3.271521
222	1	0	9.198649	3.451205	-3.271521
223	1	0	9.566919	-1.885797	-3.478458
224	1	0	9.566919	-1.885797	3.478458
225	1	0	3.565672	-10.738935	0.000000
226	8	0	7.552822	0.805551	-6.702144
227	8	0	0.239961	-8.605342	-6.642388
228	8	0	-1.314073	8.668629	-6.583648
229	1	0	2.394497	-9.830623	-6.358839
230	1	0	2.394497	-9.830623	6.358839
231	1	0	0.622873	10.233814	-6.356436
232	1	0	0.622873	10.233814	6.356436
233	1	0	8.500471	0.944307	6.821761
234	1	0	-0.651145	-8.235219	6.624283
235	1	0	-2.120443	8.139962	6.543869
236	1	0	-2.120443	8.139962	-6.543869
237	1	0	8.500471	0.944307	-6.821761
238	1	0	-0.651145	-8.235219	-6.624283
239	7	0	-7.741473	1.489027	3.147543
240	7	0	-5.580616	-0.429144	3.392548
241	7	0	-7.434975	-2.705348	3.374845
242	7	0	-9.605038	-0.789111	2.976282
243	6	0	-8.932710	2.162069	3.016036
244	6	0	-8.612371	3.557061	2.963919
245	6	0	-7.250577	3.686799	3.061487
246	6	0	-6.687704	2.371913	3.192910
247	6	0	-5.335159	2.033406	3.331211
248	6	0	-4.837019	0.719469	3.430368
249	6	0	-3.416856	0.414345	3.582228
250	6	0	-3.318265	-0.938454	3.608823
251	6	0	-4.679569	-1.454305	3.490965
252	6	0	-4.971787	-2.830266	3.519691
253	6	0	-6.252284	-3.392833	3.492005
254	6	0	-6.593279	-4.787504	3.546862
255	6	0	-7.957832	-4.893390	3.457002
256	6	0	-8.498235	-3.573226	3.321355
257	6	0	-9.831282	-3.224745	3.130150
258	6	0	-10.346690	-1.934593	2.993885
259	6	0	-11.775365	-1.635539	3.009673
260	6	0	-11.878317	-0.281980	2.999720
261	6	0	-10.511617	0.230263	2.979061
262	6	0	-10.199819	1.590358	2.964622
263	1	0	-9.344810	4.345477	2.854421
264	1	0	-6.670697	4.597082	3.044202
265	1	0	-2.623882	1.144365	3.656697
266	1	0	-2.428662	-1.542940	3.712380
267	1	0	-5.872315	-5.585693	3.638092

268	1	0	-8.552253	-5.797067	3.456067
269	1	0	-10.540339	-4.047032	3.123271
270	1	0	-12.568548	-2.372694	3.031208
271	1	0	-12.774146	0.326649	3.011436
272	1	0	-11.030706	2.287983	2.917815
273	7	0	-7.434975	-2.705348	-3.374845
274	7	0	-5.580616	-0.429144	-3.392548
275	7	0	-7.741473	1.489027	-3.147543
276	7	0	-9.605038	-0.789111	-2.976282
277	6	0	-8.498235	-3.573226	-3.321355
278	6	0	-7.957832	-4.893390	-3.457002
279	6	0	-6.593279	-4.787504	-3.546862
280	6	0	-6.252284	-3.392833	-3.492005
281	6	0	-4.971787	-2.830266	-3.519691
282	6	0	-4.679569	-1.454305	-3.490965
283	6	0	-3.318265	-0.938454	-3.608823
284	6	0	-3.416856	0.414345	-3.582228
285	6	0	-4.837019	0.719469	-3.430368
286	6	0	-5.335159	2.033406	-3.331211
287	6	0	-6.687704	2.371913	-3.192910
288	6	0	-7.250577	3.686799	-3.061487
289	6	0	-8.612371	3.557061	-2.963919
290	6	0	-8.932710	2.162069	-3.016036
291	6	0	-10.199819	1.590358	-2.964622
292	6	0	-10.511617	0.230263	-2.979061
293	6	0	-11.878317	-0.281980	-2.999720
294	6	0	-11.775365	-1.635539	-3.009673
295	6	0	-10.346690	-1.934593	-2.993885
296	6	0	-9.831282	-3.224745	-3.130150
297	1	0	-8.552253	-5.797067	-3.456067
298	1	0	-5.872315	-5.585693	-3.638092
299	1	0	-2.428662	-1.542940	-3.712380
300	1	0	-2.623882	1.144365	-3.656697
301	1	0	-6.670697	4.597082	-3.044202
302	1	0	-9.344810	4.345477	-2.854421
303	1	0	-11.030706	2.287983	-2.917815
304	1	0	-12.774146	0.326649	-3.011436
305	1	0	-12.568548	-2.372694	-3.031208
306	1	0	-10.540339	-4.047032	-3.123271
307	16	0	-5.965937	-2.053994	0.000000
308	6	0	-7.775858	-2.343220	0.000000
309	1	0	-8.208671	-1.864376	-0.882907
310	1	0	-8.208671	-1.864376	0.882907
311	6	0	-5.968594	-0.224491	0.000000
312	1	0	-6.501256	0.118544	-0.891066
313	1	0	-6.501256	0.118544	0.891066
314	6	0	-4.538792	0.301005	0.000000
315	1	0	-3.993897	-0.029331	-0.888318
316	1	0	-4.557163	1.396599	0.000000
317	1	0	-3.993897	-0.029331	0.888318
318	6	0	-8.053944	-3.841825	0.000000
319	1	0	-9.134614	-4.022469	0.000000
320	1	0	-7.627522	-4.322044	-0.883350
321	1	0	-7.627522	-4.322044	0.883350
322	1	0	-7.644193	0.480277	-3.206427
323	1	0	-7.503656	-1.699476	-3.261239

324	1	0	-7.644193	0.480277	3.206427
325	1	0	-7.503656	-1.699476	3.261239
